# Epigenetic Upregulation of lncRNAs at 13q14.3 in Leukemia Is Linked to the *In Cis* Downregulation of a Gene Cluster That Targets NF-kB

**DOI:** 10.1371/journal.pgen.1003373

**Published:** 2013-04-04

**Authors:** Angela Garding, Nupur Bhattacharya, Rainer Claus, Melanie Ruppel, Cordula Tschuch, Katharina Filarsky, Irina Idler, Manuela Zucknick, Maïwen Caudron-Herger, Christopher Oakes, Verena Fleig, Ioanna Keklikoglou, Danilo Allegra, Leticia Serra, Sudhir Thakurela, Vijay Tiwari, Dieter Weichenhan, Axel Benner, Bernhard Radlwimmer, Hanswalter Zentgraf, Stefan Wiemann, Karsten Rippe, Christoph Plass, Hartmut Döhner, Peter Lichter, Stephan Stilgenbauer, Daniel Mertens

**Affiliations:** 1 Cooperation Unit “Mechanisms of Leukemogenesis,” University Ulm, German Cancer Research Center, DKFZ, Heidelberg, Germany; 2 Signalling to Chromatin Laboratory, Institute of Molecular Biology, Mainz, Germany; 3 Division of Epigenomics and Cancer Risk Factors, German Cancer Research Center, DKFZ, Heidelberg, Germany; 4 Division of Molecular Genetics, German Cancer Research Center, DKFZ, Heidelberg, Germany; 5 Division of Biostatistics, German Cancer Research Center, DKFZ, Heidelberg, Germany; 6 Research Group Genome Function and Organization, German Cancer Research Center, DKFZ and BioQuant, Heidelberg, Germany; 7 Division of Molecular Genome Analysis, German Cancer Research Center, DKFZ, Heidelberg, Germany; 8 Division of Monoclonal Antibodies, German Cancer Research Center, DKFZ, Heidelberg, Germany; 9 Division of Internal Medicine III, University Ulm, Ulm, Germany; Cincinnati Children's Hospital Medical Center, United States of America

## Abstract

Non-coding RNAs are much more common than previously thought. However, for the vast majority of non-coding RNAs, the cellular function remains enigmatic. The two long non-coding RNA (lncRNA) genes *DLEU1* and *DLEU2* map to a critical region at chromosomal band 13q14.3 that is recurrently deleted in solid tumors and hematopoietic malignancies like chronic lymphocytic leukemia (CLL). While no point mutations have been found in the protein coding candidate genes at 13q14.3, they are deregulated in malignant cells, suggesting an epigenetic tumor suppressor mechanism. We therefore characterized the epigenetic makeup of 13q14.3 in CLL cells and found histone modifications by chromatin-immunoprecipitation (ChIP) that are associated with activated transcription and significant DNA-demethylation at the transcriptional start sites of *DLEU1* and *DLEU2* using 5 different semi-quantitative and quantitative methods (aPRIMES, BioCOBRA, MCIp, MassARRAY, and bisulfite sequencing). These epigenetic aberrations were correlated with transcriptional deregulation of the neighboring candidate tumor suppressor genes, suggesting a coregulation *in cis* of this gene cluster. We found that the 13q14.3 genes in addition to their previously known functions regulate NF-kB activity, which we could show after overexpression, siRNA–mediated knockdown, and dominant-negative mutant genes by using Western blots with previously undescribed antibodies, by a customized ELISA as well as by reporter assays. In addition, we performed an unbiased screen of 810 human miRNAs and identified the *miR-15/16* family of genes at 13q14.3 as the strongest inducers of NF-kB activity. In summary, the tumor suppressor mechanism at 13q14.3 is a cluster of genes controlled by two lncRNA genes that are regulated by DNA-methylation and histone modifications and whose members all regulate NF-kB. Therefore, the tumor suppressor mechanism in 13q14.3 underlines the role both of epigenetic aberrations and of lncRNA genes in human tumorigenesis and is an example of colocalization of a functionally related gene cluster.

## Author Summary

Recent results suggest that genome regions not coding for proteins are read and transcribed into RNA. While the function for the majority of the resulting non-coding RNA molecules remains unclear, some of them are termed according to their length (typically 200–2,000 nucleotides) as long non-coding RNA (lncRNA) genes that play a role in regulating the activity of target genes. In most instances, this deregulation involves changes of so-called “epigenetic” marks associated with the DNA that are inherited to the cellular progeny without changes in the DNA sequence. Here we describe an example where two lncRNA genes (*DLEU1* and *DLEU2*) are epigenetically deregulated together with a cluster of neighboring protein-coding tumor suppressor genes in almost all patients suffering from chronic lymphocytic leukemia. Such a common regulation suggests that the affected genes are involved in the same cellular pathway. In line with this notion, the 13q14.3 genes modulate the NF-kB signalling pathway, either inducing or repressing its activity. An activation of NF-kB has previously been shown to promote survival of the leukemic cells, underlining the importance of the 13q14.3 tumor suppressor locus for the pathomechanism of the disease.

## Introduction

Non-coding RNAs (ncRNA) are emerging as an important factor for the aberrant gene expression associated with cancer [1]. NcRNA genes are mostly involved in the regulation of target gene function [2]. Their mode of action varies from posttranscriptional regulation (i.e. miRNA genes) [3] to modulation of transcription *in cis* or *in trans*, either via competition or blockage mechanisms [4], by acting as chromatin organizers that target chromatin modifying factors (e.g. *HOTAIR, KCNLQT1* and *XIST*) [5]. NcRNA genes can even act as enhancers themselves [6]. However, for the vast majority of ncRNAs, the specific cellular function remains enigmatic.

**Figure 1:**
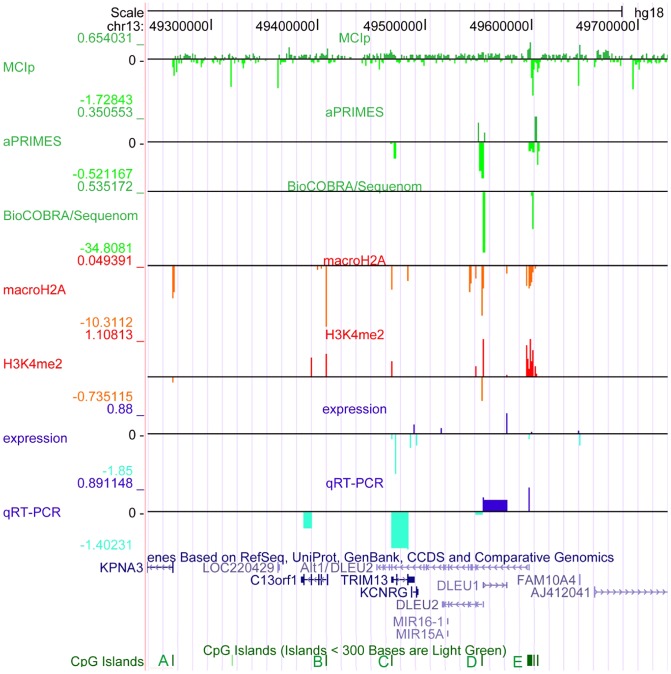
The critical region at 13q14.3 displays relaxed chromatin at the transcriptional start site (TSS) of the long non-coding RNA genes *DLEU1* and *DLEU2* in CLL cells. The critical region on 13q14.3 was analyzed for DNA-methylation (lanes 1–3), distribution of histone modifications (lanes 4, 5) and gene expression (lanes 6, 7). DNA-hypomethylation was detected in CLL cells at the transcriptional start sites of the lncRNA genes *DLEU1* and *DLEU2* variant *Alt1*. This finding coincides with enrichment of H3K4me2 and depletion of macroH2A, corroborating relaxation of 13q14.3 in CLL. While expression of the protein coding genes and *DLEU2* is decreased in CLL cells, lncRNA genes *DLEU1* and *DLEU2/Alt1* show upregulation, suggesting a direct regulation by DNA-methylation. Base pair positions on top refer to genome GRCh37 build hg18. The conserved CpG islands (A–E) are shown in green, less stringent CpG islands shown in light green. *Lane 1*: Methyl-CpG Immunoprecipitation (MCIp) allowed semi-quantitative DNA-methylation analysis in non-malignant B-cells sorted from healthy donors (n = 7) and CLL samples (n = 25). Precipitated DNA was hybridized onto a custom tiling microarray covering the 13q14.3 critical region. Depicted is the median log2 fold enrichment of CLL samples from which the median of log2 fold enrichment of healthy donor B-cell samples has been subtracted. *Lane 2*: Restriction digest-based analysis of DNA-methylation (aPRIMES) was performed in CpG islands C–E at 13q14.3 at 1 kbp resolution. Shown is the median log2 signal intensity of CLL patients from which the median of non-malignant B cell samples has been subtracted. *Lane 3*: Hypomethylation at D6 and E6 was validated by BioCOBRA and MassARRAY/Sequenome analyses (for details see Figure 2). Shown is the difference of the median percentage of methylation in CLL cells and non-malignant B cells. *Lanes 4,5*: ChIP was performed for macroH2A (lane 4) and H3K4me2 (lane 5). Precipitated DNA was quantified using qPCR. Enrichment was normalized to non-specific IgG and two control promoters (CDH12, HK2) that displayed similar enrichment for the two histone marks in CLL samples (n = 7) and peripheral blood mononuclear cells (PBMC) from healthy probands (n = 5). Enrichment was calculated as median log2 fold enrichment in precipitate vs. input for CLL samples after subtraction of enrichment from non-malignant B-cells. *Lane 6*: Gene expression profiling (GEP) was performed using bead chip arrays (Illumina) in CLL patients (n = 25) and sorted B-cells from healthy donors (n = 9). Plotted is the difference of log fold changes of CLL samples and non-malignant B cells. *Lane 7*: QRT-PCR for gene expression analysis of 13q14.3 candidate genes (for details see Figure 3).

Two long ncRNA (lncRNA) genes *DLEU1* (Gene ID: 10301) and *DLEU2* (Gene ID: 8847) map to a critical region at chromosomal band 13q14.3 that is recurrently deleted in hematopoietic and solid tumors (Figure 1) [7]–[10]. *DLEU2* splicing variants have been suggested to represent the primary transcripts (pri-miR) of *miR-15a* (Gene ID: 406948) and *miR-16-1* (Gene ID: 406950) because of their localization and coregulation [11]. *MiR-15/16* are among the most strongly and ubiquitously expressed miRNA genes in human cells [12] and appear to exert a crucial role in tumorigenesis [13]. In chronic lymphocytic leukemia (CLL), more than 50% of cases harbor a deletion of the critical region at 13q14.3 [7], [14]. Loss of 13q14.3 is also the most common aberration in the CLL precursor monoclonal B-cell lymphocytosis (MBL) [15]. The tumor suppressor mechanism at 13q14.3 is multifactorial and is likely to involve other genetic elements than *miR-15a/16-1*, since (i) knocking out *miR-15a* and *miR-16-1* in mice leads to a lymphoproliferative disease [16], but rare cases of CLL have been described where the deletion at 13q14.3 does not encompass the miRNA genes [10], [17], [18]. (ii) Deletion of a larger region at 13q14.3 including adjacent regions in addition to *miR-15a/16-1* leads to more aggressive disease in mice and humans that more frequently resembles a CLL phenotype [16], [18]–[20]. (iii) Familial CLL can be associated with deletion of *DLEU7* (Gene ID: 220107) localized more proximal in 13q14.3 than with *miR-15a/miR-16* [21].

It remains unclear how the miRNAs and the other candidate tumor suppressor genes are functionally inactivated in CLL. Sequence mutations in the miRNA genes that lead to aberrant processing from primary transcripts occur only very rarely in CLL [18], [22]–[24]. In addition, the miRNA genes may be more commonly affected by a processing defect (Allegra et al., manuscript submitted). No point mutations have been found in the other candidate genes at 13q14.3 [25]. However, in support of their role as tumor suppressors, the two miRNA genes and the other candidate tumor suppressor genes in the region are downregulated in CLL cells compared to non-malignant B-cells [10], [13], [26], [27].

Thus, epigenetic aberrations play a major role in the pathomechanism of CLL [28]–[30] and not only the genes but also regulatory sequences (e.g. CpG islands) are conserved in the mouse [31]. Accordingly, we have investigated the epigenetic features of the critical region at 13q14.3 in detail to dissect the underlying regulatory network.

Interestingly, two genes in the vicinity of the critical 13q14.3 region are imprinted (*RB1*, Gene ID: 5925 and *HTR2A*, Gene ID: 3356) [32], [33]. Parental imprinting is a mechanism where epigenetically regulated lncRNA genes control the expression of genes *in cis*. Similar to an imprinting mechanism, we recently found a complex epigenetic regulatory mechanism that involves asynchronous replication timing and monoallelic expression in non-malignant B-cells isolated from healthy donors [34], [35]. In addition, the copy of an epigenetic aberration to the homologous chromosome could account in CLL for the observed high incidence of loss of heterozygosity at 13q14.3 without loss of genetic material or the occurrence of mutations [10], [36]. Incomplete inactivation by epigenetic markers could also explain the frequent occurrence of genetic loss of the second copy of 13q14.3 in clonal evolution [34], [37]. In summary, these findings together with the transcriptional deregulation in CLL cells made it very likely that the function of the tumor suppressor mechanism at 13q14.3 is lost through epigenetic aberrations.

We therefore characterized the epigenetic makeup of 13q14.3 in a thoroughly selected cohort of CLL patients (n = 143, see Table S1 for patient characteristics), and found significant DNA-demethylation of two specific sequences within conserved CpG islands at the transcriptional start sites (TSS) of *DLEU1* and *DLEU2/Alt1* (ENST00000425586). This epigenetic aberration was correlated with transcriptional deregulation of the neighboring candidate tumor suppressor genes. Such a coregulation *in cis* of several tumor suppressor genes points to a functionally related gene cluster that is involved in the same cellular pathway. In support of this view we found that the 13q14.3 candidate tumor suppressor genes *KPNA3* (Gene ID: 3839), *RFP2* (Gene ID: 10206) and *C13ORF1* (Gene ID: 57213) are positive regulators of NF-kB activity. In addition, we performed an unbiased screen of 810 human miRNAs and showed the *miR-15/16* family of genes to be the strongest inducers of NF-kB activity. As one major function of NF-kB in CLL has been shown to be prevention of apoptosis [38]–[40], our findings contrast with the supposed role of the 13q14 genes as tumor suppressor genes. Based on these results it will be tempting to dissect the exact molecular link between 13q14.3 and NF-kB in CLL. In summary, the tumor suppressor mechanism at 13q14.3 is orchestrated by two epigenetically controlled lncRNA genes regulating a cluster of genes that impact on NF-kB.

## Results

### CLL cells display epigenetic marks at 13q14.3 associated with relaxed chromatin and transcriptional activation

For a comprehensive characterization of the epigenetic make-up of the critical region at 13q14.3 in CLL cells, DNA-methylation of the whole region (from *ITM2B* Gene ID: 9445 to *DLEU7*) was quantified in primary patient and healthy proband samples (Table S1 for patient and Table S2 for healthy proband characteristics). In addition, the CpG islands of the candidate genes were analyzed for changes in histone modifications. Applying five different techniques for detection and quantification of DNA-methylation [41]–[45], we found that two regions displayed significantly different DNA-methylation patterns in CLL cells compared to non-malignant B-cells (Figure 1, lanes 1–3; Figures S1, S2). The differentially methylated regions are localized within the CpG islands D and E at the transcriptional start sites of the lncRNA genes *DLEU1* (region “D6”) and the *DLEU2* variant *Alt1*, respectively (region “E6”; Figure 1, Figure S1 for validation and S2A, S2B for detail). In a region of chromosomal band 3q25.33 that shows a genetic makeup similar to 13q14.3, no aberrant DNA-methylation could be detected (Figure S2C, S2D). Also no differential DNA-methylation was found in CLL at the retinoblastoma tumor suppressor gene *RB1* at 13q14.3 that has been implicated in the pathomechanism of the disease [16], [18], or the *DLEU7* gene (Figure S2D, S2E) [46]. To corroborate the finding of a relaxed chromatin conformation in CLL, the CpG islands C, D and E were analyzed for the presence of histone modifications that correlate with open chromatin and active transcription (dimethylation of H3K4, “H3K4me2”) [47] or with epigenetic mechanisms leading to transcriptional inactivation (macroH2A) [48]. In line with a more relaxed chromatin in CLL cells as compared to non-malignant cells, H3K4me2 showed significantly more enrichment, while less chromatin was precipitated that carried the macroH2A modification (Figure 1, lanes 4 and 5). Therefore unexpectedly, active chromatin marks were detected in CLL cells at 13q14.3 instead of repressive epigenetic marks that are characteristic for tumor suppressor inactivation.

### Epigenetic aberrations at 13q14.3 are independent of clinical and genetic characteristics and affect 95% of all CLL patients

**Figure 2:**
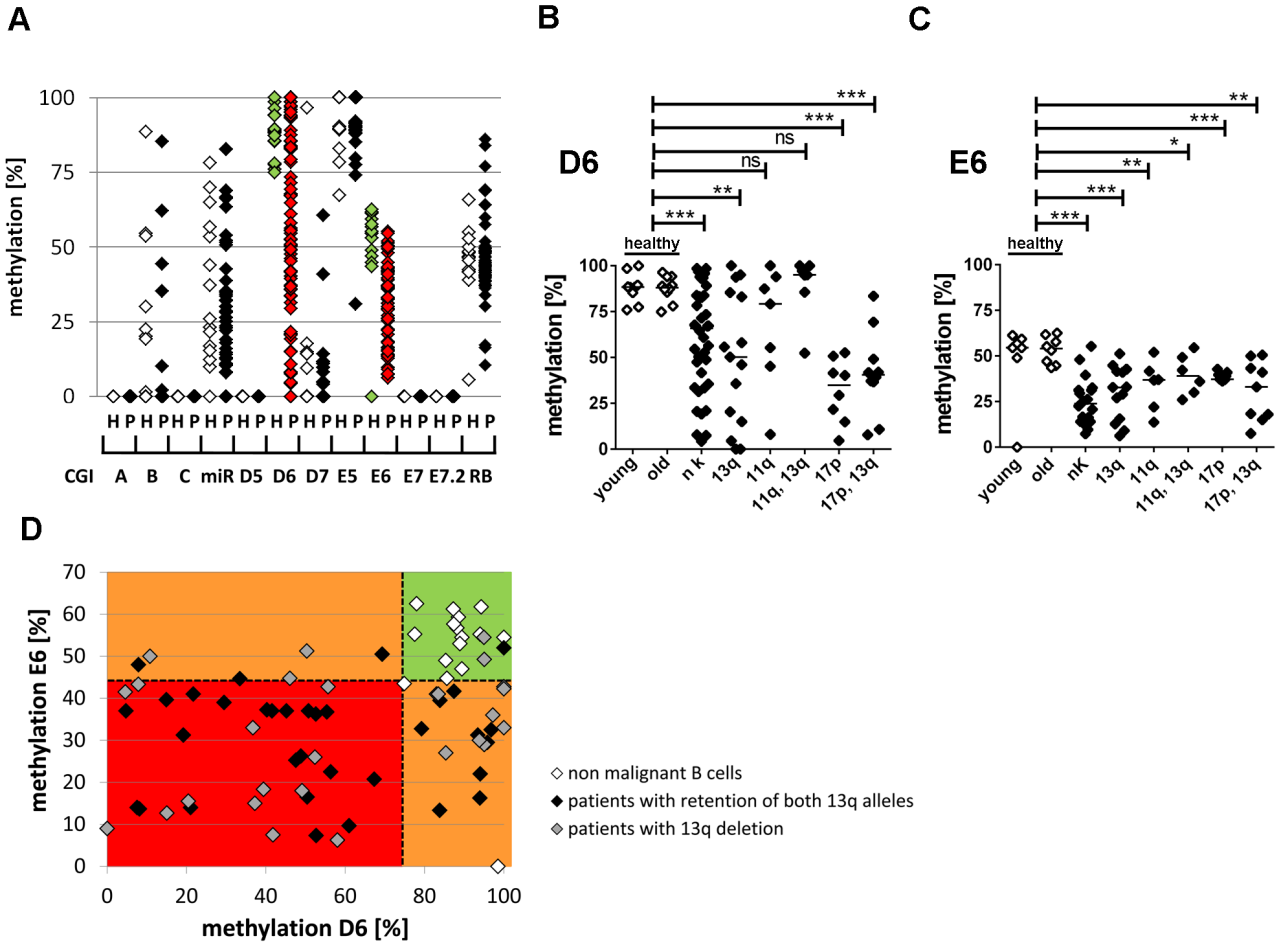
Hypomethylation at the TSS of the large ncRNAs affects the majority of CLL patients independent of 13q gene dosage and IGHV mutation status. (A) For validation of DNA-hypomethylation at different CpG islands in 13q14.3 (x-axis; for localization see figure 1), quantitative measurement with BioCOBRA and MassARRAY (“Rb”) technology was performed in B-cells of healthy donors (“h”, n = 15–19, white diamonds) and PBMCs of CLL patients (“p”, n = 47–82, black diamonds; “miR” = 308 bp fragment spanning miR-15a; “Rb” = intragenic CpG island). (B, C) CLL patients with different genomic aberrations show different degree of hypomethylation that is distinct from healthy probands younger (“young”) or older (“old”) than 45 years. DNA-methylation was quantified with BioCOBRA (D6) and MassARRAY (E6). Analyzed were patients with normal karyotype (“nk”), deletion of 13q14.3 (“13q”), deletion of 11q22-q23 (“11q”), deletion of 17p (“17p”) or combinations thereof. Statistics were performed using Wilcoxon rank sum test (*** p<0.001, ** p 0.001 to 0.01, * 0.01 to 0.05 and ns not significant p>0.05). (D) DNA-methylation of D6 or E6 is aberrant in 58/61 CLL patients. Black diamonds: Patient samples with two copies of the critical region in 13q, grey diamonds: samples with deletion of one copy of 13q14.3, white diamonds: healthy donor samples. Dashed lines represent lowest levels of methylation of non-malignant B-cells.

In order to test whether aberrant DNA-methylation is independent of prognostic and cytogenetic characteristics and thus a unifying feature of CLL, we analyzed a larger cohort of CLL patients (Figure 2, Table S1). DNA-hypomethylation was independent of 13q14.3 gene dosage and was also not a result of the advanced age of the patients (Figure 2B and 2C, compare age-matched controls; characteristics are listed in Table S2; for Mann-Whitney Rank Sum Test see Table S6). Interestingly, DNA-methylation was significantly retained in CLL cells with a deletion of 11q22-q23 covering the *ATM* (Gene ID: 472) gene, and the most pronounced loss of DNA-methylation was found in patients with a deletion of TP53 (Gene ID: 7157; Figure 2B and 2C). It can be speculated that the DNA-damage repair function of the ATM kinase could be involved in aberrant DNA-demethylation [49] or that a defect in 11q could be epistatic to loss of function of 13q14.3 [50], but this needs to be shown in further analyses. Finally, levels of DNA-methylation were not significantly correlated with mutation status of the immunglobulin heavy chain variable segment genes (IGHV), an important prognostic marker in CLL (Figure S3A) [51], or with overall survival (Figure S3B and S3C), implying that DNA-hypomethylation is present in CLL patients from all prognostic subgroups. In order to complement the single time point analyses of DNA-methylation with assessment of the dynamic changes over time, we analyzed peripheral blood mononuclear cell (PBMC) samples collected from patients at different time points during the course of the disease (Figure S3D). Intriguingly, 4/10 CLL PBMC samples (P7-P10) displayed more DNA-hypomethylation at 13q14.3 than would be expected from the content of CLL cells within the PBMC sample. These findings suggest that DNA-demethylation at 13q14.3 could be an ongoing process in CLL and should also be studied as a marker for imminent disease progression.

In summary, DNA-methylation at 13q14.3 was aberrantly lower in 58 of 61 patients (95%) compared to non-malignant B-cells (Figure 2D), proposing that DNA-hypomethylation at 13q14.3 seems to be a universal feature of CLL.

### 13q14.3 genes are a coregulated gene cluster whose expression correlates with DNA-methylation in CLL cells

**Figure 3:**
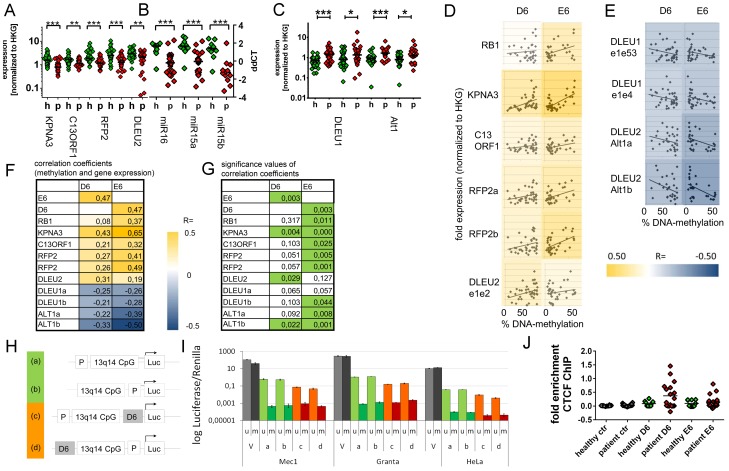
Downregulation of 13q14.3 candidate tumor suppressor genes and upregulation of lncRNA genes *DLEU1* and *DLEU2/Alt1* correlates with DNA-methylation. (A–C) Expression of the protein-coding genes *KPNA3, C13ORF1* and *RFP2*, the miRNA genes *miR-15a* and *miR-16-1* and the lncRNA transcripts from genes *DLEU1, DLEU2* and *alternative transcript Alt1* were quantified with qRT-PCR in CLL cells from patients with retention of both copies of 13q14.3 (“p”, n = 34; see Table S1) and compared to B-cells from healthy donors (“h”, n = 20). Mean expression is indicated by a black horizontal line. “HKG” = housekeeping genes, average of *PGK, LMNB1* and *PPIA*. (D, E) DNA-methylation levels correlate with transcript levels of genes in 13q14.3: While expression (y-axis) of candidate tumor suppressor genes is directly correlated to DNA-methylation of regions D6 and E6 (x-axis; left panel), lncRNA genes are anti-correlated (right panel). Pearson correlation coefficients are color-coded, blue = negative, yellow = positive correlation. (F) Pearson coefficients of correlation of gene expression (rows) and DNA-methylation at D6 and E6 (columns), colour coded (see legend). DLEU1a = exon1 to exon4; DLEU1b = exon 1 to exon 2; ALT1a/b = exon1, two different amplicons (G) Significance values of Pearson correlation coefficients (t-distribution), values p<0.05 are coded green. (H) The bidirectional promoter of *DLEU1* and *DLEU2* and the flanking CpG island was cloned into pCpGL luciferase vector, either including D6 (orange) or excluding D6 (green). (I) Constructs depicted in (H) were either methylated *in-vitro* using SSsI methylase (m, dark bars) or left unmethylated (u, light bars) and subsequently transfected into Mec1, Granta519 and HeLa cells and luciferase activity quantified. Grey: Luciferase CMV expression vector (V) not containing CpGs was used as negative control, green: excluding D6 region, orange: including D6 region. Error bars signify standard deviation of 2 independent experiments, each performed in duplicate. No changes were detected for E6 (Figure S4G–S4J). (J) Binding of the DNA-methylation sensitive chromatin reader CTCF was enriched in CLL samples both in the D6 and E6 regions compared to non-malignant B-cells. CTCF-bound chromatin was immunoprecipitated and quantitated with qPCR. Specific binding was shown by quantification of a sequence not bound by CTCF 2 kb upstream of the DM1 insulator (“ctr”). Statistics were performed using Wilcoxon rank sum test (*** p<0.001, ** p 0.001 to 0.01, * 0.01 to 0.05 and ns not significant p>0.05).

Next we investigated the functional impact of the epigenetic aberrations in 13q14.3. As reported previously, the protein-coding and the miRNA candidate tumor suppressor genes (including their host gene *DLEU2*; [11]) in the critical region are downregulated in CLL cells (Figure 3A and 3B) [26]. In contrast, the lncRNA genes *DLEU1* and variant *DLEU2/Alt1* that display DNA-hypomethylation at their 5′ ends are significantly upregulated in CLL cells (Figure 3C). To exclude bias caused by the influence of more complex interrelations e.g. by deletion of the critical region, we focused on samples with retention of both copies of 13q14.3. We found a significant inverse correlation of gene expression of the lncRNA genes *DLEU1* and the *DLEU2* variant *Alt1* [52] with DNA-methylation levels in regions D6 and E6 that are localized at their transcriptional start sites. The Pearson correlation coefficient for *DLEU2/Alt1b* with D6 was R = −0.33 (p = 0.022) and for *DLEU1* with E6 the coefficient R = −0.28 (p = 0.044; see Figure 3E, for correlation coefficients see panels F, G). This suggests the direct regulation of *DLEU1* and *DLEU2/Alt1* by DNA-methylation. In contrast, expression of the protein-coding genes in the region and the miR-15a/-16-1 host gene *DLEU2* were positively correlated with DNA-methylation levels (Figure 3D; correlation coefficients F, G), suggesting an indirect regulation by DNA-demethylation e.g. via the lncRNA genes. Levels of mature *miR-15a* and *miR-16* showed no significant correlation with DNA-methylation levels, probably because they are subject to additional posttranscriptional deregulation (Allegra et al., manuscript submitted). Differences in DNA-methylation supposedly reflects differential binding of transcription factors, and we comparatively analysed the sequences at D6 and E6 for binding motifs of transcription factors by comparing it to the TRANSFAC database using PATCH (PatchTM public 1.0, http://www.gene-regulation.com/cgi-bin/pub/programs/patch/bin/patch.cgi). Intriguingly, a number of transcription factor binding motifs are present both in the D6 and E6 sequence, further suggesting that these sequences might be regulated by similar pathways (Table S3).

### DNA-demethylating agent 5-aza-2′-deoxycytidine upregulates 13q14.3 genes in cultured cells

In order to understand how DNA-demethylation of D6 and E6 would impact on transcriptional deregulation of 13q14.3, we first tested 16 cell lines for presence of DNA-methylation at 13q14.3 (Figure S4A, S4B) and whether DNA-demethylation results in transcriptional deregulation of 13q14.3 candidate genes. Only Jurkat cells showed DNA-methylation in both loci and could be DNA-demethylated both at D6 or E6 (Figure S4A–S4F). Downregulation of the protein-coding candidate genes detected in CLL cells could not be reproduced in Jurkat cells *in-vitro*, probably because either the cellular system (T-cells) or the treatment does not faithfully reproduce the complex *in-vivo* situation (Figure S4F). Interestingly, the levels of mature miR-15a and miR-16 also remained unchanged, which is in line with a recent report where incubation of CLL cells with a inhibitor of histone deacetylases (HDACi) led to upregulation of *miR-15a* and *miR-16-1* in only 35% of patient samples [53]. These findings suggest that the miRNA genes are regulated at the post-transcriptional level in the majority of CLL patients (Allegra, manuscript submitted). However, as expected we could show that both lncRNA genes *DLEU1* and *DLEU2/Alt1* were upregulated in Jurkat cells upon DNA-demethylation *in-vitro* (Figure S4F), underlining that their transcriptional activity depends on levels of DNA-methylation.

### DNA-demethylation regulates lncRNA expression *in vitro* and correlates with CTCF binding in a subset of CLL patients

In order to test the functional relevance of the hypomethylated DNA-sequences, their impact on the expression of luciferase reporter constructs was quantified. The two sequence elements D6 and E6 completely lost their capacity to activate transcription when they were *in-vitro* DNA-methylated (Figure 3H and 3I, Figure S4G–S4J), which is in line with an upregulation of the lncRNA genes in CLL cells upon DNA-demethylation. In addition, inclusion of the non-methylated D6 sequence led to a transcriptional inhibition of the reporter construct in all three cell lines analyzed, suggesting that transcriptional inhibitors might bind to the sequence element (Figure 3H and 3I). In contrast, no changes in transcription were found when the E6 element was included (Figure S4G–S4J), suggesting that either no transcription factors would bind to that sequence or that the reporter system did not faithfully reproduce the *in-vivo* situation. Both could be the case if E6 would represent an element of higher order chromatin e.g. a boundary element. Such an element could be bound by CTCF protein (Gene ID: 10664), which insulates active chromatin from heterochromatic gene deserts [30] reminiscent of the region distal to *DLEU1* that is gene-poor. In addition, CTCF has a central role in transcriptional control exerted by ncRNA genes *in cis*, probably by segregating regulatory elements like enhancers and promoters [54], and its binding to DNA is sensitive to DNA-methylation [55]. In addition, CTCF binding sites were predicted to be localized close to or within D6 and E6 (Figure S4K). We therefore tested CTCF binding at 13q14.3 using ChIP-qPCR, and in fact CTCF binds to E6 and D6 in a subset of CLL cells but not in sorted B-cells from healthy donors (Figure 3J). Therefore, CTCF is a candidate for modulating transcription at 13q14.3 *in cis* in a subset of CLL cells.

### 13q14.3 lncRNA genes do not bind to chromatin

In order to further delineate the regulatory mechanism of the lncRNA genes *DLEU1* and *DLEU2/Alt1*, we tested whether they exert their function by binding to chromatin. As expression levels of *DLEU1* and *DLEU2/Alt1* were too low for direct visualization of the lncRNA transcripts using RNA-FISH, we used RNA-seq of RNA bound to chromatin [56] that was isolated from murine embryonic stem cells, HeLa and U2OS cells. However, compared to the other genes localized in the critical region, no significant enrichment of *DLEU1* or *DLEU2* transcripts was found to be bound to chromatin (Figure S5). It is therefore unlikely that *DLEU1* or *DLEU2* exert their function by binding to chromatin, but rather regulate the neighboring cluster of candidate tumor suppressor genes by divergent transcription (see Discussion). This coregulation of the 13q14.3 genes implies that they are also functionally related, e.g. that the respective gene products are involved in similar cellular processes. To understand which common pathway is targeted by the 13q14.3 candidate genes, we analyzed their gene function.

### The epigenetically coregulated 13q14.3 genes form a functional cluster of interacting genes that modulate NF-kB signalling

For most of the 13q14.3 candidate genes, the associated molecular function remains unclear. Examples are *miR-15a* and *miR-16-1*, for which a role in regulation of the cell cycle has been shown [16], [57]–[59]. Interestingly, for these miRNA genes and for several additional gene products at 13q14.3, an involvement in the NF-kB pathway has been postulated: *miR-15a* and *miR-16-1* (inducing NF-kB) [60] and *DLEU7* (repressing NF-kB) [61] modulate this central signalling pathway. For *KPNA3*, whose loss leads to an expansion of hemocytes in Drosophila [62], binding of the NF-kB DNA-binding subunit p65/RELA (Gene ID: 5970) has been reported, suggesting a NF-kB inductive role [63]. Because of this suggestive functional link of the 13q14.3 gene cluster, our further experiments focused on their involvement on NF-kB signalling.

### *MiR-15/miR-16* gene family is the strongest inducer of NF-kB in the miRNome

**Figure 4:**
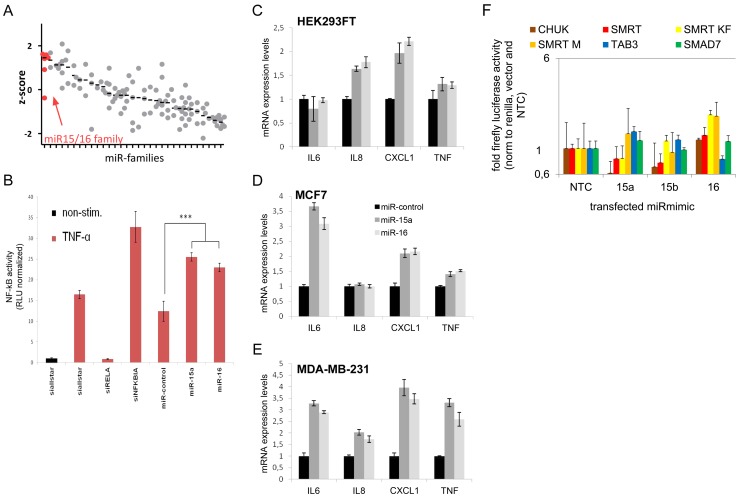
*MiR-15/miR-16* family is the strongest inducer of NF-kB. (A) NF-kB induction by miRNA families (see Table S5 for complete list) was measured using NF-kB luciferase reporter assay. Median z-scores are indicated by black horizontal lines. Each dot represents one miRNA-mimic. (B) Transfection of miRNA-mimics *miR-15a* and *miR-16* induces NF-kB activity as compared to unspecific control siRNA. Error bars represent standard deviation of triplicate measurements. (C–E) Upon transfection of miRNA mimic *miR-15a* and *miR-16*, expression of NF-kB target genes measured by qRT-PCR is induced in HEK293FT, MCF7 and MDA-MB-231 cells. (F) 3′UTRs of genes involved in the modulation of NF-kB activity and shown to be *miR-15a* and/or *miR-16* targets were cloned into luciferase reporter plasmids. After co-transfection with miR-mimics into HEK cells, firefly luciferase activity was measured and normalized to renilla luciferase activity and empty luciferase plasmid as transfection and background control, respectively, and to no-template control (“NTC”). Data is depicted on a logarithmic scale to balance lower and higher activity compared to control, error bars show standard error of the mean of three independent experiments.

First we tested whether *miR-15a* and *miR-16* modulate NF-kB with an unbiased whole genome miRNA (miRNome) screen and measured NF-kB activity with a luciferase reporter assay [64]. Of 810 miR-mimics transduced into HEK293 cells, the *miR-15a/miR-16* family (*miR-15a, miR-15b, miR-16, miR195, miR424, miR497*) showed the strongest induction of NF-kB of all tested miRNA families (Figure 4A). Compared to a non-specific control miRNA, transfection of *miR-15a* and *miR-16* miRmics into HEK293 cells significantly enhanced the induction of NF-kB by TNFalpha (Figure 4B). In line with this finding, NF-kB target genes like *IL6, IL8, CXCL1* and *TNFalpha* were induced in three different cell lines derived from embryonic kidney and breast cancer, albeit with different induction patterns (Figure 4C–4E), suggesting that the modulation of NF-kB by the *miR-15/16* miRNA family can occur in different tissues. Thus, in addition to their previously reported role in regulation of cell-cycle associated genes [16], [58], [65], the miR-15/-16 family of genes is capable of inducing NF-kB. As activation of NF-kB has been shown in CLL cells to prevent apoptosis [38]–[40], an inducive effect of miR-15/-16 of this pathway is difficult to reconcile with their tumorsuppressive role at least in the tissue analysed here. Therefore to validate an involvement of miR-15/-16 in NF-kB signalling we sought to identify target genes that modulate NF-kB in addition to the previously reported target genes that are associated with cell cycle progression.

### *miR-15/miR-16* family represses genes that modulate NF-kB activity

The *miR-15/miR-16* family of miRNAs has been reported to target several genes involved in NF-kB signalling: *IKKa/CHUK*, the NF-kB activating kinase itself (Gene ID: 1142) [66], *TAB3* (Gene ID:257397), an adaptor protein connecting TRAF6 with the NF-kB activating kinase TAK1 [60], and the transcriptional coregulator *NCOR2/SMRT* (Gene ID: 9612) [67]. As a control we included *SMAD7* (Gene ID: 4092) that is a predicted target of *miR-15a* (TargetScan6.2 algorithm), and a negative modulator of NF-kB activity [68] but has not been validated as a target so far. In order to delineate the molecular mode of induction of NF-kB activity by *miR-15a, miR-15b* and *miR-16*, the respective miR-mimics were cotransfected with luciferase reporter constructs containing 3′UTRs or parts of the 3′UTRs of the candidate target genes into HEK293T cells. While constructs containing 3′UTRs of genes previously reported to be targets of *miR-15a* and/or *miR-16* (*CHUK/IKKa, SMRT* and *TAB3*) showed lower luciferase activity after miRmimics-15a/-16 transfection, luciferase activity from the control reporter *SMAD7* selected using *in-silico* prediction remained constant (Figure 4F). Thus we reproduced previously reported findings on gene targets of the miR-15/miR-16 family that modulate NF-kB transcription factor activity either directly (*NCOR2/SMRT*) or via upstream kinases (*IKKa/CHUK*) or upstream adaptor proteins (*TAB3*). The strong induction of NF-kB by the miR-15/miR-16 family in our screen however suggests that additional genes are targeted by these miRNAs that are part of the NF-kB circuitry.

### *KPNA3* modulates and *RFP2* induces NF-kB in HEK293 and primary CLL cells

**Figure 5:**
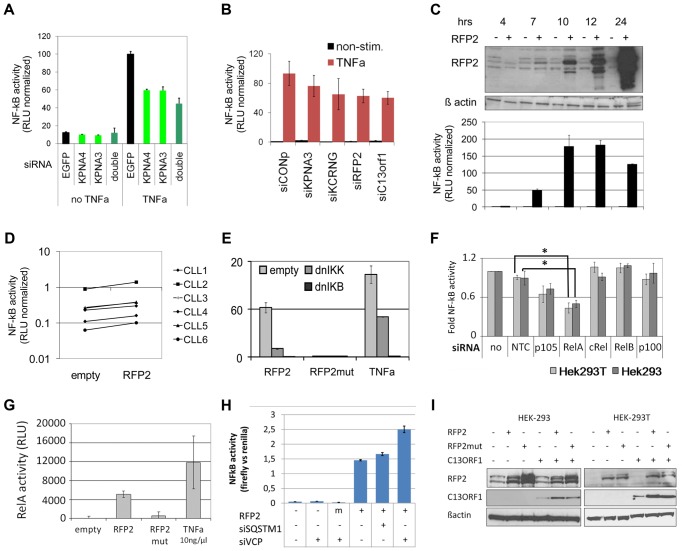
13q14.3 candidate genes are a functionally related gene cluster that modulates NF-kB signalling. (A) Knockdown of *KPNA3* and *KPNA4* (x-axis) resulted in reduced stimulation of NF-kB by TNFa in U2OS cells. NF-kB activity was measured after 24 hrs with a reporter driving luciferase expression under the control of 5 canonical NF-kB recognition sites. SiRNA directed against EGFP was used as negative control. Standard deviation of duplicate measurements is shown. (B) *KPNA3, KCNRG, RFP2* and *C13ORF1* were knocked down in HEK293T cells and activity of NF-kB was measured. As negative control, siRNA without a physiological target was used (siCONP). Error bars signify SEM of 3 independent experiments. (C) RFP2 induces NF-kB activity in HEK293T and HEK293 (Figure S6B) cells. HEK293T cells were transiently transfected with CMV RFP2 expression plasmids or empty vector and NF-kB activity measured after 4, 7, 10, 12 and 24 hrs (bottom panel). The top panel is a representative Western blot of two experiments, error bars in bottom panel represent standard deviation of triplicate measurements. (D) *RFP2* induces NF-kB activity in primary CLL cells (n = 6 patients; CLL3 is beneath CLL5). Experimental setup as in (C). (E) Induction of NF-kB by *RFP2* can be inhibited with dominant-negative IkB kinase (dnIKK) and dominant-negative IkB (dnIKB). HEK293T cells were transfected with expression vector encoding wt-*RFP2* or mutant *RFP2* in combination with dnIKK or dnIkB or empty vector, and activity of NF-kB was measured with a luciferase reporter. Inhibition of NF-kB activity by dnIKK and dnIkB was controlled by stimulation with TNFa 6 hrs after transfection and quantification of NF-kB activity after 24 hrs. (F) *RFP2* induces NF-kB activity via p65 and p105. p105, RELA (p65), cREL, RELB and p100 were knocked down in HEK293 and HEK293T cells that were transfected with *RFP2* expression plasmid. NF-kB activity was measured with luciferase reporter assay. *p<0.05 with students t-test. (G) *RFP2* induces DNA-binding of RELA. HEK293T cells were transfected with empty vector (“empty”), expression plasmid containing wildtype *RFP2* (“RFP2”), *RFP2* with mutated ubiquitin ligase activity (“RFP2mut”) or were stimulated with TNFalpha (“TNFa”). After 24 hrs, DNA-binding capacity of RELA was quantified by co-ELISA. Error bars represent standard deviation of 2 independent experiments. (H) Knockdown of *VCP* but not *SQSTM1* modulates activation of NF-KB by RFP2. *SQSTM1* and *VCP* were knocked down and plasmids for overexpression of *RFP2* and luciferase reporters detecting NF-kB activity were transfected after 24 hrs into HEK-293. While only a minor change could be observed after knockdown of SQSTM1, knockdown of VCP led to a substantial increase in the activation of NF-kB after cotransfection of RFP2. (I) Cotransfection of *RFP2* leads to stabilization of *C13ORF1* protein. HEK-293 and HEK-293T cells were transfected with wildtype *RFP2* or mutated *RFP2* lacking ubiquitin ligase activity alone or in combination with *C13ORF1*. Protein levels of *RFP2* and *C13ORF1* were monitored by Western blot. β-actin served as loading control. Experiment is representative of two independent experiments.

We confirmed that knockdown of *KPNA3* located in 13q14.3 and the family member *KPNA4* (Gene ID: 3840) located in 3q25.33 (Figure S2D) leads to a loss of inducibility of NF-kB activity by TNFalpha (Figure 5A). However, double knockdown of both genes did not lead to a full loss of NF-kB induction. In addition we analysed whether *C13ORF1, RFP2* and its bicistronic ORF *KCNRG* (Gene ID: 283518), the protein-coding genes closest to or included in the minimally deleted region, are also involved in NF-kB signalling. To this end we knocked down candidate genes from the minimally deleted region and induced NF-kB with TNFalpha (Figure 5B). Even though TNFalpha activates NF-kB via several pathways, knocking down *RFP2* and, depending on the NF-kB recognition sequence used, also *KCNRG* and *C13ORF1* led to a decrease in the activation of NF-kB (Figure 5B, Figure S6A). To further validate the role of *RFP2* in NF-kB signalling, we exogenously overexpressed *RFP2* in HEK293-T, HEK293, controlled overexpression of *RFP2* with a specific antibody we raised in guinea pig against recombinant full length *RFP2* and quantified NF-kB activity with a luciferase reporter assay (Figure 5C; Figure S6B). An induction of NF-kB was also observed when *RFP2* was overexpressed in primary CLL cells (Figure 5D). As overexpression of recombinant proteins may lead to artificial activation of NF-kB signalling, we separately overexpressed recombinant GFP as a negative control using 50 fold more plasmid than *RFP2* expression plasmid and did not observe activation of NF-kB (Figure S6C), underlining that the effect of *RFP2* is specific.

### *RFP2* activates NF-kB subunits RelA and p105 via its ubiquitin ligase activity

These findings raise the question how *RFP2* activates NF-kB. NF-kB activation is only induced 7hrs after transfection of the RFP2 expression plasmid, which is the same time point when the exogenous RFP2 protein can first be detected (Figure 5C, Figure S6B). This suggests a direct effect by overexpression of *RFP2* and excludes transcriptional induction of other factors, which would require at least an additional 1–2 hrs. The activation of NF-kB by RFP2 could be blocked by dominant negative (dn) IKK and dnIkB [69], suggesting that the effect of RFP2 takes place upstream of these factors (Figure 5E). In addition, loss of the ubiquitin-ligase activity of *RFP2* by mutating C13A [70] completely abrogated the activation of NF-kB by *RFP2* (Figure 5E). In order to identify the NF-kB component that is targeted by RFP2 activation, all DNA-binding components of the NF-kB signalling pathway were knocked down individually. Downregulation of RELA and to lesser extent of p105 (Gene ID: 4790) reduced the activation of NF-kB by RFP2 (Figure 5F). Corroborating this finding in a custom oligonucleotide-coupled ELISA (co-ELISA) [71], *RFP2* specifically induced the activity of RELA (Figure 5G).

### Activation of NF-kB by RFP2 is modulated by VCP

It has recently been shown that the RFP2 protein interacts with VCP, ATP2A2/SERCA2 [70] and SQSTM1 [72]. We therefore asked whether these proteins would be involved in the modulation of NF-kB activity by RFP2. However, knockdown of ATP2A2 and SQSTM1 did not result in enhanced activity of NF-kB (Figure 5H; Figure S6D). In contrast, knockdown of VCP substantially increased the activation of NF-kB by RFP2 (Figure 5H and Figure S6D). This finding is intriguing as VCP and SQSTM1 link RFP2 not only to endoplasmatic reticulum associated protein degradation (ERAD) and autophagy [70], [72], but also to regulation of TRAF6 [73], [74] that is involved in signalling pathways such as CD40/CD40L and TLR that are central to the pathogenesis of CLL.

### *RFP2* overexpression stabilizes C13ORF1 protein

*RFP2* is a member of the family of tripartite motif proteins (TRIM) but lacks the SPRY domain (pfam00622) common to other TRIM proteins [75]. Intriguingly, the neighboring *C13ORF1* gene has a SPRY domain, suggesting a functional interaction of RFP2 and C13ORF1. While knockdown of *C13ORF1* led to a reduction in NF-kB inducibility (Figure 5B), co-expression of RFP2 and C13ORF1 did not lead to a synergistic induction of NF-kB activity (Figure S6D). As RFP2 is an integral membrane protein [70] and requires disruptive RIPA buffer extraction for analysis, physical interaction with C13ORF1 could not be shown by pulldown experiments. However, support for the interaction of *RFP2* and *C13ORF1* proteins came from the observation that coexpression of *RFP2* stabilized expression of *C13ORF1* (Figure 5I), even though RFP2 has a destabilizing (auto-)ubiquitin ligase activity [70].

## Discussion

### The functional gene cluster at 13q14.3 is transcriptionally deregulated by hypomethylation in malignant cells

**Figure 6:**
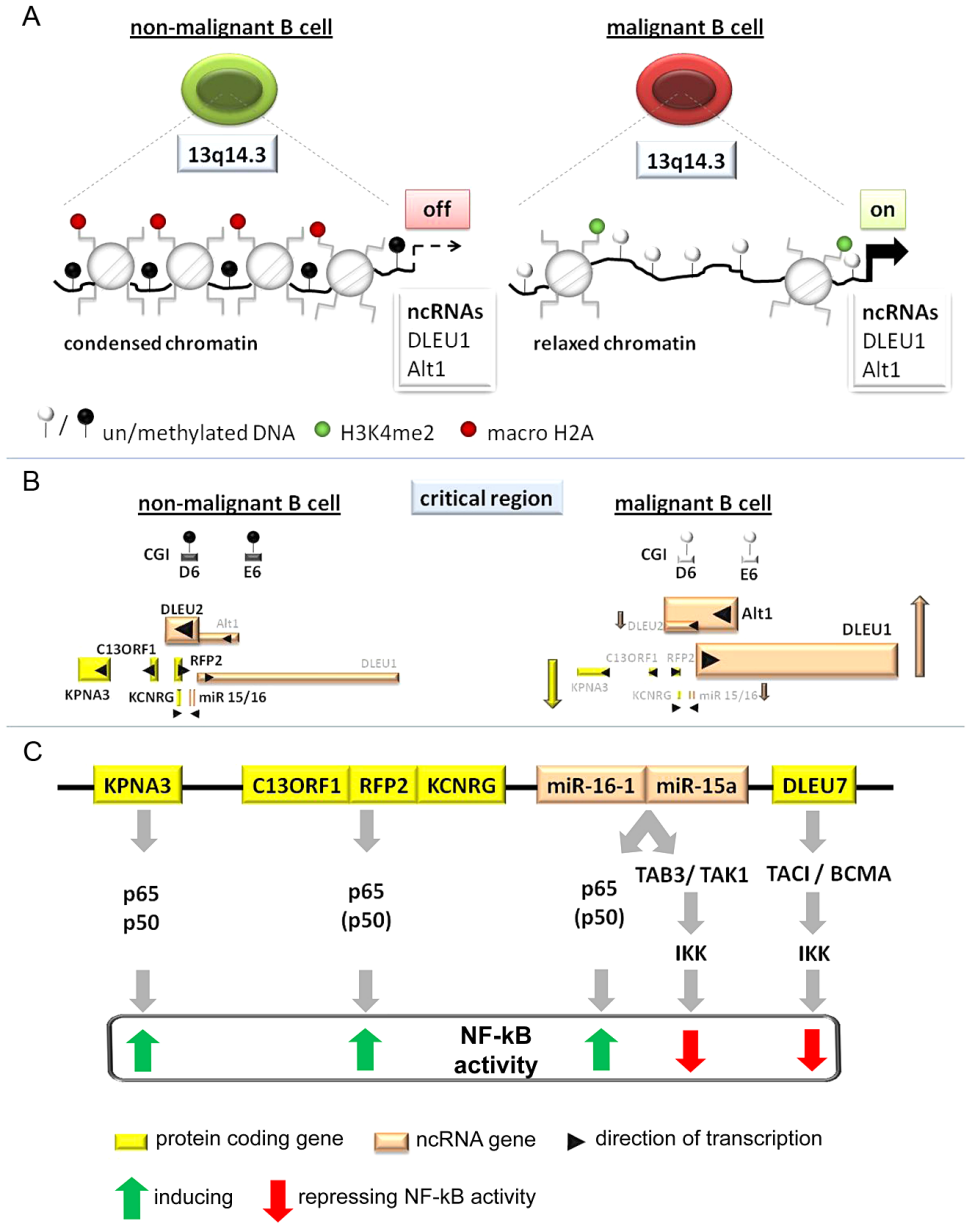
Model of the tumor suppressor mechanism localized in 13q14.3. (A) Regions in CpG islands D and E that are DNA-methylated in non-malignant B-cells (left) become demethylated in the vast majority of CLL patients (right). This coincides with relaxed chromatin characterized by absence of macroH2A and enrichment of H3K4me2 at the promoters of the lncRNA genes *DLEU1* and *DLEU2/Alt1*. (B) DNA-hypomethylation is correlated with transcriptional upregulation of splicing variants of the two lncRNA genes *DLEU1* and *DLEU2* and inversely correlated with the protein-coding genes in 13q14.3. No correlation could be found with levels of mature *miR-15a* and *miR-16*, probably because these transcripts are also deregulated by a posttranscriptional processing defect in CLL cells (Allegra, manuscript submitted). (C) Candidate genes localized in the critical region in 13q14.3 are functionally related and all modulate NF-kB signalling, albeit with different impact. Nuclear transporter KPNA3 is binding NF-kB components p65 and p50 and is therefore likely a positive regulator like e.g. RFP2. RFP2 binds to C13ORF1 and induces NF-kB activity via the canonical pathway components p50 and p65, for which its ubiquitin ligase actvity is required. The miRNA genes *miR-15a* and *miR-16-1* were identified together with other members of this miR family to be among the strongest activators of NF-kB activity. Previously they have been shown to both modulate cell cycle regulators and inhibit NF-kB via *TAB3/TAK1. DLEU7* has recently also been reported to inhibit NF-kB by binding and inactivating the *TACI/BCMA* receptors.

Several lines of evidence suggest that the tumor suppressor function of 13q14.3 distal to *RB1* is multigenic [16], [20] and is not inactivated by mutation [25], but rather by transcriptional deregulation [10], [13], [26], [27]. In line with this notion we found DNA-demethylation at the 5′ends of the lncRNA genes *DLEU1* and *DLEU2* in more than 95% of CLL patients (Figure 2D, Figure 6A). Hypomethylation in cancer cells usually coincides with chromosomal instability, which we cannot exclude for 13q14.3, or with reexpression of silenced oncogenes. In fact, a genome-wide DNA-demethylation has been observed for CLL cells [76], but the functional consequence is unclear. At 13q14.3, DNA-methylation levels comparable to non-malignant B-cells were observed at all tested loci except for the elements D6 and E6. DNA-demethylation of the regions D6 and E6 in CLL cells is directly correlated with an increase in the expression of *DLEU1* and *DLEU2* and inversely correlated with the expression of the neighboring candidate tumor suppressing protein-coding genes (Figure 6B). The expression of antisense transcripts is usually lower and is not necessarily coupled to expression of the respective sense transcripts [77], which is what we observed at 13q14.3 for the lncRNA genes *DLEU1* and *DLEU2* and the protein-coding genes.

As for the *cis*-regulatory mode of action of lncRNA genes, a direct RNA-DNA interaction has been shown to recruit repressors, which leads to changes in chromatin conformation [4], [78]. However, this option seems unlikely as for none of the transcriptional units at 13q14.3 we found substantial enrichment in the chromatin-bound RNA fraction.

A second mode of action could be competition by (i) “divergent transcription” of the ncRNA genes that recruits essential factors away from the candidate tumor suppressor genes [78], (ii) collision of transcription complexes initiated from different promotors (e.g. E6 and the DLEU2 promotor) but transcribing through the same sequences, or (iii) elongation through transcriptional regulators like enhancers/repressors that leads to the deposit of specialized epigenetic marks inhibiting transcription from the opposite strand [79]. Interestingly, D6 seems to inherently harbor transcriptional repressive properties. The described mechanisms are only dependent on the initiation of transcription and can be independent of the resulting (antisense) RNA molecule itself. The dispensable role of the *DLEU1* transcript itself is also suggested by the lack of conservation of the *DLEU1* gene sequence and its multitude of splicing variants [8]. In fact, lncRNAs involved in regulation *in cis* are in general poorly conserved, probably because these mechanisms are mostly topological [80] and thus sequence independent. A topological regulation is also suggested by the presence of a homologous region on 3q25.33 (Figure S2D) and the conservation of the orientation of the genes and CpG islands in *Mus musculus* [31]. However, it should be noted that in the mouse there is no overlap of *DLEU2* with *RFP2*, but the sequence similarity of the first exon of *RFP2* and exon 11 of *DLEU2* is conserved [9]. This suggests at least for this pair of genes the possibility of a RNAi-like regulatory mechanism. Finally, transcription in the region could be regulated by a central locus control region, organizing the intranuclear localization of 13q14.3 e.g. by binding of chromatin organizing proteins like CTCF. While DNA-methylation dependent aberrant binding of CTCF could be observed at D6 and E6 in a patient subset, more advanced experiments (e.g. 3–6C analyses) are required to assess the functional impact of changed binding properties of chromatin organizing factors such as CTCF.

Interestingly, epigenetic deregulation of lncRNA genes leading to aberrant transcription of neighboring genes occurs also in acute leukemia. The lncRNA *HOTAIRM1* for example is expressed exclusively in the myeloid lineage and controls expression of the proximal *HOXA* gene cluster [81]. Similarly, the ncRNA vault RNA2-1 (*vtRNA2-1*) in the commonly deleted region of chromosome 5q is monoallelically methylated and expressed in healthy individuals, while it is epigenetically inactivated in AML, leading to activation of NF-kB via RNA-binding protein kinase R (*PKR*, [82]). Another example resembling the molecular mechanism of the 13q14.3 locus is silencing of the tumor suppressor *WT1* by the overlapping WT1-antisense lncRNA WT1-AS, which is monoallelically expressed in non-malignant cells and becomes activated in AML by hypomethylation [83]. Thus, epigenetic deregulation of ncRNA genes seems to be a recurrent disease related phenomenon both in chronic and acute leukemias, leading to aberrant function of tumor suppressor- or oncogenes.

### The tumor suppressor genes at 13q14.3 are transcriptionally coregulated and modulate NF-kB signalling by either activating or repressing the pathway

The transcriptional activities of the 13q14.3 candidate genes all correlate with the DNA-methylation levels in the region. This co-regulation suggests that 13q14.3 genes are also functionally related, i.e. are involved in the same cellular pathways. Such clusters of genes seem more common in *drosophila* than in mammalian cells [84]. In human cells, only a subset of ubiquitously expressed genes and a small set of atypical genes is grouped together into coregulated clusters [85]. A reason for evolutionary conservation of a genetic neighbourhood of functionally connected genes is the coregulation of these genes [85]. Examples in the mammalian system are the globin gene family, groups of olfactory receptors, histone-coding genes, *HOX* genes, genes of the major histocompatibility complex and imprinted genes. In addition, most long non-coding RNA genes are involved in regulating functionally related gene clusters [2]. One major unifying scheme of these gene clusters seems to be the transcriptional activity from the same chromosomal strand [85], which has been shown for 13q14.3 [35]. Similarly, the topological organization of these gene clusters is highly conserved between mammals, which is also true for 13q14.3 [31], [50] and its homologous cluster at 3q25.33. It is therefore very likely that the 13q14.3 candidate genes are also functionally related, and we and others could show that they activate or repress the NF-kB signalling pathway (Figure 6C).

NF-kB signalling is centrally involved in the homeostasis of the hematopoietic system where it is induced in inflammation and inhibits apoptosis [86]. NF-kB signalling has already been shown to be activated in CLL cells [38]–[40], [87], where it is postulated to help in cellular survival [87]. In CLL, NF-kB is activated by the interaction with the microenvironment [39], which is crucial for the survival of CLL cells [51]. NF-kB is also activated via the B-cell receptor (BCR) that plays an important role in the pathogenesis of CLL [51]. Similarly, NF-kB is activated by interaction of TCL1 (Gene ID: 8115) and ATM in CLL [88], two genes that are coregulated in CLL cells [89] and centrally involved in the pathogenesis of CLL. In contrast, in early developmental stages of a CLL-like disease in transgenic mice, repressive p50/p50 NF-kB dimers (Gene ID: 4790) cause epigenetic lesions that even precede genetic lesions [29], suggesting that at different stages of the disease, NF-kB could play different roles.

Genome- and exome-wide analyses of CLL cells have recently shown that mutations are present in genes involved in NF-kB signalling [90], and intriguingly mutations in a NF-kB-pathway associated gene (*MYD88*, Gene ID: 4615) seem to be even enriched in del(13q) patients [91]. This is most interesting as MYD88 is required for TLR signalling via TRAF6, a protein that is bound by SQSTM1 and VCP [73], which interact with RFP2 [70], [72]. Further studies will be required to understand the molecular interplay of these proteins in full detail and especially to accommodate the unexpected induction of NF-kB activity by several genes localized at 13q14 with their tumorsuppressive function.

After recent reports have shown 13q14.3 genes to be inhibitors of NF-kB signalling [60], [61], here we demonstrate that the *miR-15a/16* cluster, *KPNA3* (and *KPNA4* from 3q25.33) and *RFP2* are positively correlated with NF-kB function (Figure 6C): the *miRNA15/16* family of genes were among the strongest inducers of NF-kB in an unbiased screen, *KPNA3* is the transporter of p65 and *RFP2* induces canonical NF-kB signalling. NF-kB activity is normally associated with an inhibition of apoptosis, and in fact has been shown to be induced in CLL cells by pro-survival microenvironmental stimulants like e.g. CD40L, BAFF; stromal cells or B-cell receptor stimulation [38], [87], [92]–[94]. However, there are a few instances where NF-kB activity can also induce apoptosis. The most relevant example is probably the negative selection of T-cells [95], where strong signalling from the T-cell receptor upon recognition of self-antigens induces apoptosis via activation of NF-kB above a certain threshold. While in B-cells negative selection is somewhat dissimilar, loss of negative selection in CLL cells would make sense considering i) the autoreactivity of CLL cells and ii) the importance of consistent BCR signalling induced by self-antigens in the pathogenesis of the disease [96]. Thus, even though within the same cell, NF-kB activity cannot be at the same time silenced and activated, the activity of NF-kB can change during the leukemogenesis of CLL and the role of the tumor suppressor mechanism in 13q14 could be required only at specific timepoints. The speculative involvement of 13q14 genes in negative B-cell selection could explain how deletion of NF-kB-inducing genes at 13q14 would lead to CLL leukemogenesis at an early timepoint, while malignant B-cells from of terminal stage CLL then exhibit increased levels of NF-kB that prevent apoptosis as has been shown previously (see above). In this respect it should be borne in mind that the functional assays quantifying the impact of 13q14 genes on NF-kB signalling performed both by us and by others depend on in-vitro experiments mostly in cell lines and not in primary cells except for *RFP2* (Figure 5D). In addition, overexpression and knockdown of 13q14 genes was performed using recombinant constructs. These experimental settings and their results might therefore not properly reflect the physiological situation, especially when looking at such finely tuned systems like NF-kB signalling. However, NF-kB has been shown to be a promising target for therapeutic intervention in CLL cells [97], [98], and further functional experiments and especially in-vivo analyses should be performed to fully understand the mechanistic link between 13q14 and NF-kB in CLL.

### Conclusion and outlook

In summary, we uncovered a cluster of functionally related genes that are coregulated by long non-coding RNA genes *in cis* and are epigenetically deregulated in malignant cells. We previously speculated that the epigenetic deregulation could explain a stepwise inactivation of the tumor suppressor mechanism [34]. This would complement the findings of clonal evolution and/or extent of 13q14 deletion being associated with a more aggressive form of CLL [18], [37], and the presence of pre-malignant stages of the disease (e.g. MBL) [15]. Further work is required to identify transcription factors binding to the demethylated regions and characterization of their intranuclear localization. It will also be of interest to test whether the observed epigenetic aberrations are present already in premalignant cells of mouse models [23], [99] or whether they constitute the aberrations that have been postulated to be present in hematopoietic stem cells of CLL patients [100].

## Materials and Methods

### Magnetic depletion and isolation of cells

Mononuclear cells were isolated from peripheral blood by density centrifugation using Ficoll (Biochrom AG) according to the manufacturer's instructions. For positive selection of CD19^+^ B-cells and CLL cells from peripheral blood, mononuclear cells (PBMCs) were labeled with CD19 MACS magnetic MicroBeads and isolated using MACS LS Column placed in the magnetic field of MACS Separator. The purity of the CD19^+^ fraction was 95%±3% (± SEM) after purification from PBMCs from healthy probands and 97%±2% for purification from PBMCs of CLL patients as measured by flow cytometry (FACSCalibur, BD Biosciences) using anti-CD19 FITC-labeled antibodies (anti-CD19 MicroBeads, Dako) that specifically binds the CD19 epitope.

### Ethics statement

Peripheral blood samples were obtained from patients after informed consent by a procedure approved by the Ethics Committee of Ulm University (approval 96/08), and peripheral blood was drawn from fully anonymised age-matched healthy probands at the german red cross (DRK) in accordance with the Declaration of Helsinki.

### QPCR (quantitative PCR) analysis and reverse transcription (RT)

Standard 20 µl qPCR reactions contained 10 µl SYBR Green mixture (Absolute QPCR SYBR Green ROX Mix, Thermo Scientific) and primers at 70 nM final concentration. Thermal cycling conditions were 15 minutes at 95°C, 40 cycles of 15 s at 95°C and 30 s at 60°C, dissociation curve 15 s at 95°C, 15 s at 60°C and heated to 95°C (within 20 minutes), held for 15 s and cooled down to 4°Ç using the 7300 Real-Time PCR system (Applied Biosystems). A standard curve, using template dilutions of HeLa and HEK293 cDNA was measured to determine PCR efficiency and allow exact quantification of template. All primers used for qPCR are listed in the Table S4. Reverse transcription of total RNA was carried out using the AffinityScript QPCR cDNA Synthesis Kit (Agilent), a reaction lacking reverse transcriptase (-RT) was included for each template where primers did not span an intron and amplification of product would have been possible from contaminating genomic DNA. For mRNA detection, Ct-values were normalized using dilution standard curves and three housekeeping genes (*PGK2, LMNB1, PPIA*) or for the miRNA genes using the ddCT method with RNU6B and SNORA73A as internal normalization controls.

### MiRNA detection

10 ng of total RNA was reverse transcribed using the miScript Reverse Transcription Kit (QIAGEN) where reactions were scaled down to 10 µl. The completed RT reactions were diluted to 50 µl with DEPC-treated water and PCR amplification for real-time quantitative analysis was performed using the miScript SYBR Green PCR kit (QIAGEN). Total reaction volume of qPCR was 20 µl, and 2 µl of the diluted RT reactions were used as template. For miRNAs custom forward primers were used to final 0.5 µM (sequences see Table S4) and primers for normalization controls RNU6B and SNORA73A were purchased from QIAGEN. The annealing temperature was 55°C.

### Cloning of 13q14.3 constructs into pCpGL vector

To study the effect of DNA-methylation *in vitro*, the regions D6 and E6 were cloned with their physiological promoter into the pCpGL vector (kind gift from Michael Rehli) [101] to investigate their impact on transcription and whether this is dependent on DNA-methylation. The promoters of the large ncRNAs *DLEU1/DLEU2* and *DLEU2/Alt1* were cloned in both directions with and without the putative regulatory elements D6 and E6. The construct containing D6 was 2 kb in size and the construct lacking D6 was 1.3 kb in size. These products could be amplified from placenta DNA (SIGMA-Aldrich) using the HotStarTaq Plus PCR system (Qiagen; cycling: 95°C: 5 min; 40 cycles of 95°C: 30 sec, 58°C: 30 sec, 72°C: 1/1.5 min; hold 10°C). Constructs containing or not containing E6 were 4000 and 3500 bp in size and amplified with the Expand High Fidelity PCR System (Roche) using PAC 372-3 from 13q14.3 as template [7]. Cycling was performed 95°C 2 min, 10× [95°C 20 s, 60°C 30 s, 68°C 4 min], 20× [95°C 20 s, 60°C 20 s, 68°C 4 min +20 s in each cycle], 68°C 7 min, hold 4°C. The desired constructs were amplified with primers containing BamH1 and SpeI recognition sites (see Table S4) and cloned into the TOPO TA cloning vector (Invitrogen). Plasmids from positive clones were digested with BamH1 and SpeI (NEB) using 3 µg TOPO-plasmid-insert-DNA or 1 µg pCpGL vector for 1 h at 37°C. The insert was isolated on a 1% agerose gel (50 min, 150 V) and extracted using QIAEX II Kit (Qiagen). For sticky end ligation a 3 times molar excess of insert over pCpGL vector backbone was used in a ligation reaction with 0.1u T4 ligase (Invitrogen) incubating 1 h at 37°C or at 16°C over night.

The ligation reaction was purified by ethanol precipitation, resuspended in 5 µl water and 1 µl was used to transform competent PIR1 E.coli cells (Invitrogen) bacterial cells via electroporation (Gene Pulser II, BIO-RAD). After electroporation in a 2 mm cuvette at 25 µF and 2.5 kV setting the pulser at 200×, transforming 50 ng DNA within a total volume of 400 µl, cells were plated on zeocin containing plates and incubated at 37°C overnight. For transient transfections, plasmids were isolated and purified using the EndoFree Plasmid Kit (Qiagen). *In vitro* methylation was performed using SssI methylase (NEB) according to manufacturer's instructions but incubating for 4 h at 37°C and adding 1 µl fresh SAM after 2 h.

### Cloning of 3′UTRs of miR target genes into pMIR-Report plasmid

To measure the impact of miR-15a, miR-15b and miR-16 on potential target genes, parts of or the whole 3′UTRs of *TAB3, CHUK, SMAD7* and *SMRT* were cloned into the vector pMIR-Report (Applied Biosystems). Sequences containing the miR target sites in the 3′UTRs of *TAB3, CHUK, SMRT “KF”* and *SMAD7* were amplified from HEK293T genomic DNA using the corresponding primers (containing restriction sites for HindIII, SpeI or SacI; see Table S4) and the PRECISOR high-fidelity DNA polymerase (BioCat) according to the manufacturers instructions. Amplified products were purified using the PCR Purification Kit from Qiagen, digested with HindIII, SpeI or SacI (FastDigest Enzymes, Fermentas) and ligated with pMIR-Report (T4 DNA Ligase, Fermentas). Plasmid backbone had been digested with the respective enzymes and purified via agarose gel extraction (Qiagen). Reporters containing just the miR target site and the respective mutated sequence (“SMRT” and “SMRTmut”) were cloned as described previously [67].

### Bisulfite conversion

1 µg of genomic DNA was converted using EpiTect 96 Bisulfite Kit or EpiTect Bisulfite Kit (Qiagen) in a GeneAmp PCR System 2700 (Applied Biosystems) with a reaction volume of 100 µl. After desulphonation, converted DNA was eluted 2 times in 20 µl prewarmed (65°C) water. Bisulfite conversion was performed on dilution series (different degree of methylation) of placenta DNA (SIGMA-Aldrich), DNA from CLL patients and from B cells of healthy individuals for quantitative methylation analysis by BioCOBRA or massARRAY as well as bisulfite sequencing.

### BioCOBRA

For BioCOBRA analysis (combined bisulfite restriction analysis with the Agilent 2100 Bioanalyzer platform, [102], bisulfite converted DNA was amplified using primers specific for converted template (see Table S4). After purification of the PCR products using Rapid PCR Purification System (Marligen Biosciences), products were digested with BstUI (NEB) over night at 60°C. Fragments were subsequently analysed with DNA 1000 LabChip (Agilent) on the Agilent 2100 Bioanalyzer. For all amplicons a calibration curve was measured with defined mixtures of methylated and unmethylated DNA (Figure S1A). Fully unmethylated DNA was generated by whole genome amplification (REPLI-g Kit, Qiagen), and after purification (QIAamp DNA Mini Kit, Qiagen), half of the amplified and purified DNA was *in vitro* methylated using SssI methylase (NEB).

### MassARRAY

Mass-spectrometric methylation analysis was performed using MassARRAY (Sequenom) analysis according to [42] for the potential regulatory element E6 within 13q14.3, because lack of a BstUI recognition site precluded BioCOBRA. The target gene regions were amplified by PCR (see Table S4) after sodium-bisulfite conversion of template DNA using primers specific for converted template. In this amplification, reverse PCR primers were tagged with the T7 recognition sequence for reverse transcription. Deoxynucleotides in the PCR reaction were dephosphorylated using shrimp alkaline phosphatase (SAP) at 37°C for 20 min followed by 5 min heat inactivation of SAP at 85°C. Making use of the T7 recognition sequence, a single-stranded RNA copy of the template was generated by *in vitro* transcription. The produced RNA was cleaved specifically at Uracil by RNase A. The cleavage products were analyzed using matrix-assisted laser desorption ionization – time of flight (MALDI-TOF) mass spectrometry in a final elution volume of 27 µl. Cleavage product signals with a 16 Da shift (or a multiple thereof) represent methylation events; signal intensity was correlated with the degree of DNA-methylation.

### Bisulfite sequencing

Bisulfite converted DNA (EpiTect Bisulfite Kit, Qiagen) was amplified using primers specific for converted DNA. After PCR purification (Rapid PCR Purification System, Marligen Biosciences) the product was cloned into pCR2.1-TOPO vector and subsequently transformed into One Shot Mach1-T1 competent E. coli cells (Invitrogen). Positive clones were selected by colony PCR using M13 primers; cycling: 95°C: 12 min; 40 cycles of 95°C: 30 sec, 55°C: 30 sec, 72°C: 1 min; 72°C: 7 min; hold 10°C. PCR products of the expected size were purified (Marligen Biosciences) and sequenced (BigDye Terminator v3.1 Cycle Sequencing Kit, Applied Biosystems) using M13 forward primer with the ABI Prism 3100 Genetic Analyzer 3130xl (Applied Biosystems). Cycling: 96°C: 1 min; 25 cycles of 96°C: 10 sec, 52°C: 5 sec, 60°C: 2 min; hold 10°C. The sequencing reactions were purified with the DyeEx 96 Kit or DyeEx 2.0 Spin Kit (Qiagen) to remove non-incorporated nucleotides. Analysis of sequences was performed using MethTools (http://genome.imb-jena.de/methtools/).

### Array-based profiling of reference-independent methylation status (aPRIMES)

aPRIMES was performed according to [45] using 500 ng genomic DNA that was digested using 10 U MseI (NEB) for 3 h. The MseI-fragments were then subjected to linker mediated PCR using primer ddMse11 and primer Lib1 at an initial annealing temperature of 65°C that was shifted down to 15°C with a ramp of 18°C/min (MWG, Ebersberg, Germany) and ligation using T4-DNA-Ligase (10 U, Roche) was performed at 15°C overnight. Half of the resulting ligated MseI fragments were digested with the restriction enzyme McrBC (NEB) for 8 h and the other half of was digested with two methylation-sensitive endonucleases, HpaII and BstUI 3 h each. Proteinase K (Invitrogen) was used for digestion before amplification using Expand Long Template system (Boehringer) and Lib1 primer in a MWG thermo cycler; cycling: 72°C: 3 min 20 cycles (94°C: 30 s, 62°C: 30 s, 72°C: 90 s), 72°C: 10 min. The PCR products were recovered by ethanol precipitation and DNA was eluted in 30 µL 0.1× TE, pH 8. *In* vitro methylated CpG islands from rice were used as positive controls for methylation and 10 pg were spiked in DNA samples used for aPRIMES to control methylation and methylation-sensitive digestion. Mitochondrial CpG island clones that were present in the original library were used as controls for unmethylated and allelically/partially methylated CGIs.

### Methyl-CpG-immuno-precipitation (MCIp)

Genomic DNA (2 µg) isolated from CD19 sorted B cells of either CLL patients or healthy individuals was immuno-precipitated using recombinant MBD2–Fc fusion protein [103]. DNA was homogenized through a 22G needle and fragmented to a mean size of 400–500 bp using ultrasonication (2×30pulses, 24 s, 10% amplitude, Bioruptor, Diagenode). 30 µg of MBD-Fc protein was coupled to SIMAG protein-A magnetic beads (Chemicell) 3 h at 4°C in TBS. After completing MBD-Fc protein binding to the magnetic beads, precipitation of the sonicated sample DNA was performed in low salt buffer for 3 h at 4°C. Fractionated elution from the beads was performed using buffers A–F with increasing salt concentrations. In order to ensure complete elution of methylated DNA, elution with buffer F was repeated once. The collected fractions were desalted using the MinElute Kit (Qiagen) and eluates were diluted 1∶10 and analyzed for control genes (*SNRPN*, *ZAP70*; primer sequences see Table S4) with qPCR. Samples were subsequently processed for array hybridization.

### Chromatin immunoprecipitation (ChIP)

For ChIP 1–5×10^7^ viably frozen CD19-sorted B cells from either CLL patients or healthy individuals were washed once with DMEM medium, taken up in 1 ml PBS and formaldehyde cross link was performed at a final concentration of 1% for 10 min at RT while rotating. Cross-linked samples were sonicated in 300 µl SDS lysis buffer (1% SDS, 10 mM EDTA, 50 mM Tris/HCl pH 8.1, 167 mM NaCl, protease inhibitors) 8 times 30 seconds on/off at high amplitude using a Bioruptor (Diagenode). The sonicated material was diluted 1∶10 with dilution buffer (0.01% SDS, 1.2 mM EDTA, 16.7 mM Tris/HCl pH 8.1, 1.1% TritonX100, protease inhibitors), subjected to 1 h preclearing with 30 µl of salmon sperm saturated protein A/G agarose beads (Millipore). Precleared chromatin samples were incubated over night with either 5 µg specific antibody (CTCF, H3K4me2, macroH2a1.2, Millipore), or 5 µg normal IgGs (Santa Cruz) at 4°C. Antibody bound chromatin was precipitated by adding 50 µl of salmon sperm saturated protein A/G agarose beads 4 h at 4°C and unspecifically bound material was removed by washing with low salt buffer, high salt buffer, LiCl buffer and two times with TE buffer. Cross link was reversed over night at 65°C and RNaseA (30 min, 37°C) as well as ProteinaseK digest (2 h, 45°C) was performed before purification of precipitated DNA using GFXTM PCR DNA and Gel Band Purification Kit (GE Healthcare). Precipitation efficiency was analyzed by qPCR for positive and negative control regions (for primers see Table S4) on antibody and control IgG precipitated fractions and expressed as percentage of input DNA using a calibration curve for quantification. Predicted CTCF binding sites were identified at http://www.essex.ac.uk/bs/molonc/binfo/ctcfbind.htm.

### Array production and hybridization

Microarrays were either produced by spotting PCR-amplified 1 kbp fragments from the promotors of *RFP2*, *DLEU1* and *DLEU2/Alt1* (CpG islands C, D and E) for aPRIMES. For MCIp arrays were custom designed (eArray, Agilent) to tile promotors −3.8 to +1.8 kbp from the transcriptional start sites of the region chr13:47702475–49164179 (*ITM2B – EBPL*) and complete tiling of the region chr13:49265143–50317955 (*C13ORF1 – DLEU7*; GRCh37 hg18). 60 bp oligonucleotides were designed with 30 bp nonoverlapping spacing. The resulting 9863 oligonucleotides were combined with 10 bp linker sequence and had an average melting temperature of 70.43°C. For custom arrays, the 13q14.3 oligonucleotides were complemented with the Agilent normalization group (1262) and replicate group (4626) oligonucleotides. Labeling of ChIP and MCIp samples was performed using the BioPrime Total Genomic Labeling System (Invitrogen). For CTCF ChIP samples the precipitate was labeled using Cy5 and the input was labeled with Cy3. For the MCIp samples only the elutions from the high salt fraction were labeled, the common reference (T cell pool) was labeled with Cy3 and the CLL/healthy donor sample with Cy5. In order to predict labeling efficiency, the samples were measured at the wavelengths A260, A320, A555, A650, A750, and the following equations were used to determine the yield:

Cy3: DNA amount [ µg] (A260–A320)*50*0.04, Dye incorporation (A555–A650)/0.15*40

Cy5: DNA amount [ µg] (A260–A320)*50*0.04, Dye incorporation (A650–A750)/0.24*40

The hybridization of the MCIp samples was performed according to protocol number G4170-90012 for Agilent Microarray Analysis of Methlylated DNA Immunoprecipitation version. 1.0. The hybridization of the ChIP samples was performed as described in the protocol number G4481-90010 for Agilent Mammalian ChIP-on-chip version 10.1 applying the instructions given for the 4× format.

### Aza-deoxy-cytidine treatment

Cell lines were seeded at a density of 5×10^6^ in 4 ml of the appropriate medium in 6-well plates. After 24 hrs, they were treated with a final concentration of 1.5 µM 5-Aza-2′-deoxycytidine (Sigma-Aldrich) or the respective amount of DMSO solvent in the control reaction for 6 days with daily medium and drug replacement.

### Chromatin fractionation for sequencing of chromatin-bound RNA

The chromatin fraction of RNA was prepared from isolated nuclei after shearing in a Covaris sonicator (Covaris, Inc.). The sample was then centrifuged and the soluble chromatin was loaded on a sucrose gradient as described [104]. Fractions containing DNA fragments > 5000 bp (equivalent to 25 nucleosomes with a 200 bp nucleosome repeat length) were pooled. RNA was phenol/chloroform-extracted after proteinase K and DNase I treatments and RNA-sequencing was performed. After rRNA depletion, RNAs were subjected to metal ion catalyzed cleavage to sizes between 60–200 nucleotides with the Ambion RNA fragmentation reagents. Libraries for Solexa sequencing were generated according to the standard protocol for mRNA (Illumina) that comprised first strand cDNA synthesis, second strand cDNA synthesis, end repair, addition of a single A base and adapter ligation. PCR products were size excised from low melting agarose gels (200–400 bp range) and phenol extracted. Sequencing was performed on the Illumina GAIIx platform at the sequencing core facilities of the EMBL, DKFZ and BioQuant in Heidelberg, Germany. Initial RNA sequence analysis was performed with the Bioconductor (http://www.bioconductor.org) package for the R statistical programming language to assess the read quality and to produce a reads coverage file. The integrative genomics viewer (http://www.broadinstitute.org/igv) was used to visualize the coverage file and the RefSeq genes (NCBI). Reads were aligned on the GRCh37/hg19 (2009) assembly version of the human genome reporting unique hits without mismatches and with and without trimming of the 3′ and 5′ ends. Data is available at ArrayExpress (www.ebi.ac.uk/arrayexpress), Experiment name: lncRNAs at 13q14.3; ArrayExpress accession: E-MTAB-1335 (U2OS) and E-MTAB-582 (HeLa).

### Transfection and knockdown

All cell lines were cultured according to DSMZ (www.dsmz.de) recommendations. Adherent cell lines were transiently transfected with indicated constructs using the Nanofectin Kit (PAA). A half confluent flask of cells was transfected according to manufactures instructions using 5 µg of DNA for a 25 cm^2^ flask and 8 µg for a 75 cm^2^ culture vessel. Suspension cell lines were transfected according to the Nucleofection protocol of Amaxa (Lonza). For each cell line 2×10^6^ cells were transfected using 5 µg plasmid DNA (2 µg pmax GFP as transfection control) and 100 µl Nucleofector solution. The whole procedure was performed following manufacturer's instructions and preparing the 12-well plates with 1.5 ml prewarmed medium. For Nucleofection the protocol A-023 was used. Gene transcripts were knocked down using either the Universal probe library system (Roche Diagnostics) and validated by q-RT-PCR to be below 30% of siCONP-treated cells. siRNAs were also synthesized using the Silencer siRNA construction kit (Ambion) and modified according to [105], or validated siRNAs were ordered from Applied biosystems. For simultaneous transfection of plasmids and miR-mimics (“miRVANAs”) or miR-inhibitors (both Life Technologies, Darmstadt, Germany) into HEK293T cells, Lipofectamine2000 (Invitrogen, Karslruhe, Germany), Hyperfect (Quiagen) or miRus transit-LT1 (Geneflow) was used. 4×10^5^ cells/well were seeded in 24 well plates and transfected according to the manufacturers instructions after 24 h using 0.5 µg Plasmid and 10 pmol miR-mimics or miR inhibitors, respectively. For single transfection of 10 pmol miR-mimics or miR-inhibitors, the same protocol was used and the cells harvested after 24 h for Western blot or expression analysis. Transfection efficiency was tested by transfection of pmaxGFP (Lonza, Cologne, Germany) or siGlo (Dharmacon, Darmstadt) and subsequent detection by flow cytometry.

### Luciferase assay

#### RFP2, C13ORF1, and D6/E6 elements in pCpGL

4×10^5^ cells were seeded within a 6-well plate in 2 ml medium and co-transfected with 250 ng pRL-CMV-Renilla and 750 ng of the luciferase containing constructs using the appropriate transfection reagent after 24 h. For the constructs containing/not containing the D6/E6 element, equimolar amounts of construct were transfected. After 20–24 h, the cells were stimulated where indicated with 20 ng/ml TNFa for 10 min. The medium of the cells was removed and to each well 250 µL of Passive Lysis buffer (Promega) was added and incubated at RT for 15 min on a shaker. 15 µL of the cell lysates were placed into a 96-well Nunclon White plate while Luciferase buffer and Renilla buffer were brought to RT. The plates were measured with the Glomax Luminometer (Promega) applying the following parameters for both injectors: injection volume: 75 µl; delay between injection and measurement: 0.4 s; integration time: 5 s. The obtained values for Firefly Luciferase activity were normalized to Renilla readings to standardize for transfection efficiency.

#### miRNA screen

For the NF-kB reporter assay, HEK293FT cells were transfected in 96-well plates with siRNAs or miRNA-mimics from Dharmacon (Lafayette, CO, USA) together with 75 ng of the NF-κB reporter 3×KBL (kind gift from George Mosialos, Aristotle University, Thessaloniki, Greece) and 2,5 ng of pMIR-REPORT β-gal vector (Ambion, Austin, TX, USA). 48 h after transfection cells were stimulated with TNF-α (20 ng/ml), and luciferase activity was measured after another 5 h. β-galactosidase activity was used for normalization, and measured by beta-glo Luminescent Assay Kit (Promega, Madison, WI, USA).

#### Impact of miR-15a, miR-15b, and miR-16 on the expression of TAB3, IKKa/CHUK, SMRT, and SMAD7

Parts or complete 3′UTRs of *TAB3, CHUK, SMRT* and *SMAD7* cloned into pMIR-Report Luciferase Plasmid were transfected into HEK293T cells. To this end, 4×10^5^ cells were seeded in the wells of 24 well plates with 0.45 µg of pMIR-Report, 0.05 µg TK Renilla and 10 pmol of either miR-15a-3p, miR-15a-5p, miR-15b-5p or miR-16 miR-mimics or miR-inhibitors (Life Technologies, Darmstadt, Germany), respectively. The cells were harvested after 24 h and firefly and renilla luciferase activity measured using the Dual-Luciferase Reporter Assay (Promega) with injection volumes of 50 µl for LARII and Stop & Glo solution. Luminescence was measured using the LB940 Multimode Reader Mithras (Berthold Technologies).

### Generation of RFP2 antibodies in guinea pigs

Specific antibodies were raised by immunizing guinea pigs with recombinant RFP2 S154-E264 10×His-tagged purified proteins. Guinea pigs were immunized for the first time at 8 weeks of age with 100 µg of protein per animal diluted 1∶1 with Complete Freud's adjuvant (Sigma). Subsequently animals were immunized monthly with 100 µg of protein per animal diluted 1∶1 with Incomplete Freud's adjuvant (Sigma). During this process, serum was taken periodically by heart-punction. After 24 months animals were bled. Blood was collected in Vacutainer Blood collection tubes (BD) left at RT for 1 hr and blood cells sedimented by spinning at 2000 rpm 1 hr at RT. After sedimentation, serum was collected and aliquoted for storage at −80°C or complemented with 0.02% sodium azide as preservative and kept at 4°C. The polyclonal antibodies were tested against purified recombinant protein in Western blot analysis and against protein over-expressed in mammalian cells by Western blot analysis and immunofluorescence.

### Statistical methods

Overall survival curves were estimated by the Kaplan-Meier method. Logrank tests were used for comparing survival distributions between groups. Wilcoxon rank sum tests were used to test for differences in expression or methylation distributions between two groups. For significance of Pearson correlation coefficients, t-distribution was calculated with t = r/sqrt[(1−r∧2)/(N−2)]. For assessment of statistical significance, test results with p-values p<0.05 were considered to be statistically significant.

## Supporting Information

Figure S1 BioCOBRA and MassARRAY analysis allows exact and robust quantification of DNA-methylation. (Related to Figure 1, Figure 2, and Figure 3.) (A) Increasing amounts of *in vitro* methylated DNA were added to non-methylated DNA and subsequently analyzed with BioCOBRA for several regions at 13q14.3 (“A” to “E7.2”) or with MassARRAY for E6. Except for “B”, “miR” and “E6”, a strict correlation between DNA-methylation and the quotient of undigested vs total amplicon was observed. (B) Correlation coefficients (R∧2). (C) DNA-methylation of the region D6 was quantified in B-cells of four healthy donors (“H1”–“H4”) and seven CLL patients (“P1”–“P7”) using BioCOBRA (black bars). For validation, bisulfite sequencing of 15–20 clones per sample was performed. Depicted is the percentage of methylated CpGs of the whole bisulfite-PCR amplified fragment was calculated (grey bars) as well as the percentage of methylated CpGs of the BstUI sites that were addressed in the BioCOBRA assay (white bars). (D) DNA-methylation of the region E6 was analyzed in B-cells of three healthy donors (“H1”–“H3”) and four CLL patients (“P1”–“P4”) using MassARRAY (black bars) and bisulfite sequencing of 15–20 clones per sample. Shown is the percentage of methylated CpGs of the whole fragment (grey bars). No BstUI site was located in this fragment.
(PDF)

Figure S2 Details of the transcriptional start sites of *DLEU1* (A), *DLEU2/Alt1* (B) and the homologous region on 3q25.33 (C), (D) a comparison of 13q14 and 3q25 and details of *RB1* (E) and *DLEU7* (F). (Related to Figure 1.) (A) D6 is hypomethylated in CLL patients compared to non-malignant B-cells as measured by aPRIMEs and BioCOBRA. MCIp for unknown reasons did not faithfully represent hypomethylation detected by aPRIMES and BioCOBRA. (A, B) For details see legend to Figure 1. (C) At the homologous region in chromosomal band 3q26 that harbors *miR-15b* and *miR-16-2, TRIM59* and *KPNA4*, DNA-methylation was measured by MCIp and transcriptional activity by gene expression profiling. Only minor differences could be detected between CLL samples and non-malignant B-cells. Positions refer to genome build hg18. (D) Regions of 13q14.3 and 3q25.3 have similar coding potential. Performing pairwise alignment of all protein coding genes from chr13:50,139,149–51,673,000 and chr3:158,760,141–160,445,393, a striking protein sequence similarity of a subset of protein coding genes (green boxes) was found. TRIM proteins: 57% similarity, e value: 1e-65; ARL proteins: 62% similarity, e value: 2e-53; KPNA proteins 92% similarity, e value: 0. Similarly, sequence alignment of hsa-mir-16-1 and hsa-mir-16-2 showed 68,9% identity of a 90 bp overlap and alignment of hsa-mir-15a and hsa-mir-15b showed 56,1% identity of a 98 bp overlap. It can therefore be speculated that both regions arose from duplication and inversion. “cen” centromeric, “tel” telomeric. (E) DNA-methylation was measured at an internal CpG island reported to correlate with imprinting of RB1 by regulating transcription of an alternative transcript [33]. Differences in DNA-methylation can only be observed in patients with del13q, possibly caused by gene dosage effects. (E) The median log 2 fold methylation of 12 tiling array oligos spanning the CpG island at the transcriptional start site of DLEU7 (chr 13:50315373–50316150, hg18) were compared among B cells from healthy donors (n = 7), CLL patients with retention of both 13q copies (n = 6) and CLL patients with deletion of one 13q allele (n = 7).
(PDF)

Figure S3 DNA methylation of 13q14.3 sequences does not correlate with IGHV mutations status and has no prognostic impact on overall survival, but changes during the clinical course of the disease. (Related to Figure 2.) (A) Scatterplots of methylation levels of CpG D6 and CpG E6 in the low risk patient group (normal karyotype and sole 13q deletion) comparing IGHV mutated samples with unmutated samples. There are no statistically significant differences in methylation levels between IGHV mutated and unmutated groups (D6 mutated: n = 17, unmutated n = 29; p = 0.1907 and E6, mutated: n = 13, unmutated n = 29; p = 0.2907, Wilcoxon rank sum test). (B, C) Kaplan-Meier estimates of overall survival of CLL patients (cohort composition see Table S1) comparing subgroups with different methylation levels (quartiles) of CpG D6 (A) and CpG E6 (B). In the analysed patient cohort, no statistically significant differences could be detected in overall survival distributions between subgroups (logrank test), suggesting that DNA-demethylation at 13q14.3 is common to all CLL. (D) Analysis of changes in DNA-methylation in D6 over time in CLL PBMC samples. Left panel: Exemplary result for 6/10 patients where changes in methylation in the PBMC sample (red line) correlated with the changes in the content of CLL cells in the sample (blue line) as expected. Content of CLL cells was identifed from the percentage of CD5/CD19 double positive cells of all peripheral blood lymphocytes. Other panels: Four patients showed disproportional loss/gain of DNA-methylation, suggesting correlation of DNA-methylation with the clinical course of the disease. Right panel: In one patient, DNA-demethylation at 13q14.3 was directly associated with the clinical course of the disease. “P” = patient, “T” = therapy. “*” = progressive disease.
(PDF)

Figure S4 *In vitro* manipulation of DNA-methylation of D6 and E6 regions confirms functional relevance for gene expression. (Related to Figure 3.) (A, B) Haematopoetic and non-haematopoetic cell lines where tested for basal DNA methylation levels in D6/E6 using COBRA (A, n = 16) and MassARRAY analysis (B, n = 17) respectively. COBRA was controlled using *in vitro* methylated (“m”) and *in vitro* amplified non-methylated (“um”) Granta-519 genomic DNA as control and a plasmid containing several BstuI restriction sites. Jurkat, Raji and HEK cells carried methylation at both the D6 and E6 region. Only Jurkat cells showed full methylation at the D6 region and 70% methylation at the E6 region similar to B cells from healthy donors. Thus, only Jurkat cells were suited to study the impact of D6 and E6 methylation on the expression levels of 13q14.3 genes after treatment with 5-aza-2′-deoxycytidine. (C–F) DNA-demethylation of Jurkat cells *in-vitro* leads to an upregulation of 13q14.3 genes, but not of miRNA genes. Regions D6 (C) and E6 (D) that are differentially methylated in CLL patients and a CpG island reported to modulate *RB1* expression (E) become demethylated in Jurkat cells upon 5-aza-2′-deoxycytidine treatment. (F) This demethylation leads to an increased expression of genes localized in the critical region with the exception of the miRNA genes that are post-transcriptionally regulated (Allegra, manuscript submitted). Gene expression was measured as in Figure 3. (G) The promoter of *DLEU2/Alt1*, the flanking CpG island E and the region E6 were cloned into the pCpGL luciferase vector. (H–J) Constructs depicted in G were either methylated *in-vitro* using SssI methylase (“m”) or left unmethylated (“um”) and subsequently transfected into HeLa, Granta519 and Mec1 cells. Promoter activity in HeLa was very low, suggesting that essential functional elements are missing in non-hematopoietic cells. In general, luciferase activity was lower than for the D6 constructs (Figure 4F and 4G), possibly because of the larger size of the constructs (7.9 and 7.4 kbp (E6) vs 5.9 and 5.2 kbp (D6)). Blue boxes mark constructs cloned in the physiological orientation. (K) Schematic representation of regions analyzed for CTCF enrichment by ChIP-qPCR. Red lines represent predicted CTCF binding sites (http://bsproteomics.essex.ac.uk:8080/bioinformatics/ctcfbind.htm). Green boxes represent amplicons of ChIP qPCR and blue boxes represent D6 and E6 elements, respectively. Genomic locations are depicted on top of each panel.
(PDF)

Figure S5 RNA–seq of chromatin-bound RNA shows no enhanced binding of *DLEU1* and *DLEU2* to chromatin. (Related to Figure 3). (A) In HeLa and U2OS cells, *DLEU1* and *DLEU2* do not show higher enrichment in the chromatin-bound RNA fraction when compared to the neighboring protein-coding genes and to total RNA. This suggests that they do not act via binding to chromatin. Localization of *DLEU1* and *DLEU2* is represented by the red box (top panel). Lines denote genes, blue boxes denote exons, arrows give direction of transcription. Blue bars represent numbers of reads, normalized to the highest peak whose number of reads is given at the left. (B) RNA-seq of chromatin-bound lncRNA genes used as controls. LncRNA reported to bind to chromatin show either specific enrichment in the chromatin-bound fraction (*XIST* and *Tsix* in mouse embryonic stem cells) or binding of specific sequences to chromatin (*MALAT1* and *TERC* in HeLa and U2OS cells).
(PDF)

Figure S6 13q14.3 candidate genes are involved in NF-kB signaling. (Related to Figure 4.) (A) Knockdown of 13q14.3 candidate tumor suppressor genes results in reduced activation of NF-kB by TNFa. *KPNA3, KCNRG, RFP2* and *C13ORF1* were knocked down in HEK293T cells and activity of NF-kB was measured after 24 hrs with a second reporter construct containing 5 synthetic NF-kB recognition sites. As negative control, a siRNA without physiological target was used (siCONP). Error bars signify SEM of 3 experiments. (B) *RFP2* induces NF-kB activity in HEK293 cells. HEK293 cells were transiently transfected with CMV RFP2 expression plasmids (“+”) or empty vector (“−”) and NF-kB activity was measured after 4, 7, 10, 12 and 24 hrs (bottom panel). The top panel shows a representative Western blot of two experiments, error bars in bottom panel represent standard deviation of triplicate measurements. (C) Induction of NF-kB is not due to ectopic overexpression of protein. EVSAT, HEK293T and HeLa cells were transfected with increasing amounts of expression plasmids containing *RFP2* or *GFP*. GFP-Fluorescence visualized by microscopy validated functionality and efficiency of transfection of the GFP plasmid (not shown). NF-kB activity was measured with luciferase reporter assay (for detailed description see Figure 6). Already 100 ng of *RFP2* expression plasmid induced NF-kB activity, while no or little activity was induced with 5 µg of *GFP* expression plasmid. (D) Transfection of *C13ORF1* expression plasmids into HEK293 and HEK293T cells alone or in combination with RFP2 or RFP2mut expression plasmids does not result in additional activation of NF-kB activity as measured by luciferase reporter assay. Error bars represent standard deviation of 3 independent experiments. (E) Knockdown of *ATP2A2* and *SQSTM1* does not modulate activation of NF-kB after transfection of RFP2, but knockdown of *VCP* substantially increases activation of NF-kB by *RFP2*. *ATP2A2/SERCA2*, *SQSTM1* and *VCP* were knocked down and plasmids for overexpression of *RFP2* and luciferase reporters detecting NF-kB activity were transfected after 24 hrs into HEK-293. While no or only a minor change could be observed after knockdown of *ATP2A2/SERCA2* and *SQSTM1*, respectively, knockdown of *VCP* led to a substantial increase in the activation of NF-kB after cotransfection of *RFP2*. Error bars depict variation of two independent experiments.
(PDF)

Table S1 Characteristics of CLL patient cohort (n = 143).
(PDF)

Table S2 Characteristics of healthy donor cohort (n = 43).
(PDF)

Table S3 Transcription factors whose binding motifs are present in the D6 and/or E6 element were predicted using PATCH pattern search for transcription factor binding sites selecting a lower score boundary of 87.5.
(PDF)

Table S4 Primer sequences.
(PDF)

Table S5 miRNA families tested for induction of NF-kB, ranked according to their induction of NF-kB. (Related to Figure 4.)
(PDF)

Table S6 Mann-Whitney Rank Sum Test for differential methylation. (p-values).
(PDF)
