# Supplementary material for: Epigenetic Upregulation of lncRNAs at 13q14.3 in Leukemia Is Linked to the In Cis Downregulation of a Gene Cluster That Targets NF-kB
Source: PLoS Genet. 2013 Apr 4;9(4):e1003373. doi: 10.1371/journal.pgen.1003373 (PMC3616974; doi:10.1371/journal.pgen.1003373)
Supplement: Figure S1 — BioCOBRA and MassARRAY analysis allows exact and robust quantification of DNA-methylation. (Related to Figure 1, Figure 2, and Figure 3.) (A) Increasing amounts of in vitro methylated DNA were added to non-methylated DNA and subsequently analyzed with BioCOBRA for several regions at 13q14.3 (“A” to “E7.2”) or with MassARRAY for E6. Except for “B”, “miR” and “E6”, a strict correlation between DNA-methylation and the quotient of undigested vs total amplicon was observed. (B) Correlation coefficients (R∧2). (C) DNA-methylation of the region D6 was quantified in B-cells of four healthy donors (“H1”–“H4”) and seven CLL patients (“P1”–“P7”) using BioCOBRA (black bars). For validation, bisulfite sequencing of 15–20 clones per sample was performed. Depicted is the percentage of methylated CpGs of the whole bisulfite-PCR amplified fragment was calculated (grey bars) as well as the percentage of methylated CpGs of the BstUI sites that were addressed in the BioCOBRA assay (white bars). (D) DNA-methylation of the region E6 was analyzed in B-cells of three healthy donors (“H1”–“H3”) and four CLL patients (“P1”–“P4”) using MassARRAY (black bars) and bisulfite sequencing of 15–20 clones per sample. Shown is the percentage of methylated CpGs of the whole fragment (grey bars). No BstUI site was located in this fragment. (PDF) [file pgen.1003373.s001.pdf]

A

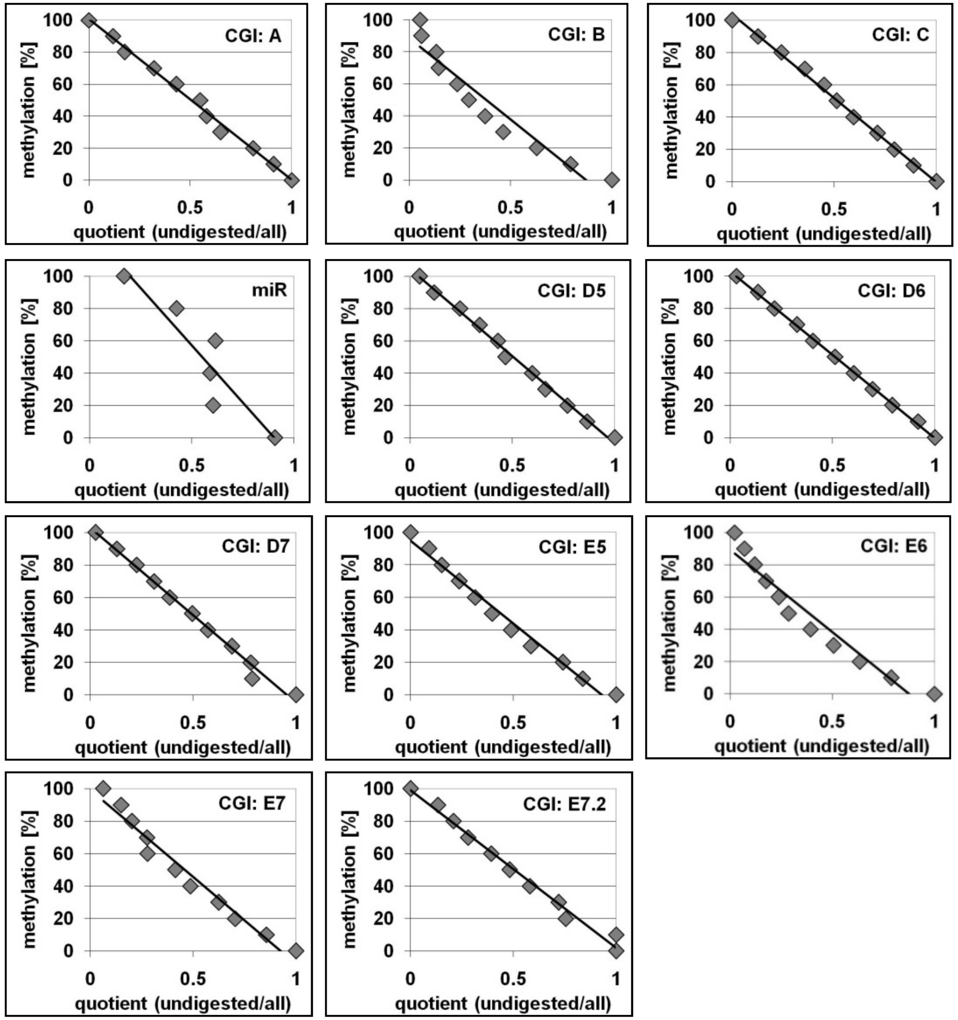

B

fragment	R^2
A	0.993
B	0.917
C	0.996
miR	0.849
D4/5	0.995
D6	0.997
D7	0.991
E5	0.984
E6	0.936
E7	0.994
E7.2	0.967

Figure S1 related to Figures 1, 2 and 3: BioCOBRA and MassARRAY analysis allows exact and robust quantification of DNA-methylation.
(A) Increasing amounts of *in vitro* methylated DNA were added to non-methylated DNA and subsequently analyzed with BioCOBRA for several regions at 13q14.3 („A“ to „E7.2“) or with MassARRAY for E6. Except for “B”, “miR” and “E6”, a strict correlation between DNA-methylation and the quotient of undigested vs total amplicon was observed. (B) Correlation coefficients (R^2).

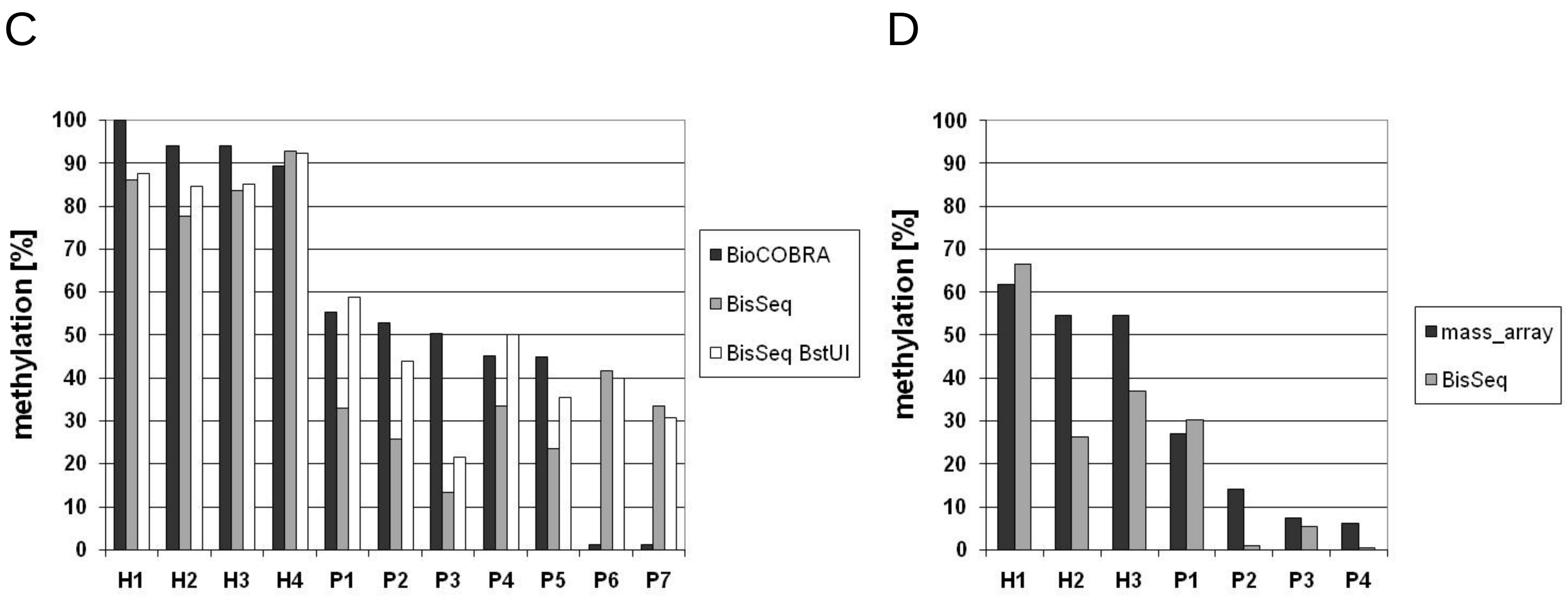

Figure S1 related to Figures 1, 2 and 3: BioCOBRA and MassARRAY analysis allows exact and robust quantification of DNA-methylation.

(C) DNA-methylation of the region D6 was quantified in B-cells of four healthy donors (“H1”-”H4”) and seven CLL patients (“P1” – “P7”) using BioCOBRA (black bars). For validation, bisulfite sequencing of 15-20 clones per sample was performed. Depicted is the percentage of methylated CpGs of the whole bisulfite-PCR amplified fragment was calculated (grey bars) as well as the percentage of methylated CpGs of the BstUI sites that were addressed in the BioCOBRA assay (white bars). (D) DNA-methylation of the region E6 was analyzed in B-cells of three healthy donors (“H1”-”H3”) and four CLL patients (“P1” – “P4”) using MassARRAY (black bars) and bisulfite sequencing of 15-20 clones per sample. Shown is the percentage of methylated CpGs of the whole fragment (grey bars). No BstUI site was located in this fragment.
