# Supplementary material for: Epigenetic Upregulation of lncRNAs at 13q14.3 in Leukemia Is Linked to the In Cis Downregulation of a Gene Cluster That Targets NF-kB
Source: PLoS Genet. 2013 Apr 4;9(4):e1003373. doi: 10.1371/journal.pgen.1003373 (PMC3616974; doi:10.1371/journal.pgen.1003373)
Supplement: Figure S3 — DNA methylation of 13q14.3 sequences does not correlate with IGHV mutations status and has no prognostic impact on overall survival, but changes during the clinical course of the disease. (Related to Figure 2.) (A) Scatterplots of methylation levels of CpG D6 and CpG E6 in the low risk patient group (normal karyotype and sole 13q deletion) comparing IGHV mutated samples with unmutated samples. There are no statistically significant differences in methylation levels between IGHV mutated and unmutated groups (D6 mutated: n = 17, unmutated n = 29; p = 0.1907 and E6, mutated: n = 13, unmutated n = 29; p = 0.2907, Wilcoxon rank sum test). (B, C) Kaplan-Meier estimates of overall survival of CLL patients (cohort composition see Table S1) comparing subgroups with different methylation levels (quartiles) of CpG D6 (A) and CpG E6 (B). In the analysed patient cohort, no statistically significant differences could be detected in overall survival distributions between subgroups (logrank test), suggesting that DNA-demethylation at 13q14.3 is common to all CLL. (D) Analysis of changes in DNA-methylation in D6 over time in CLL PBMC samples. Left panel: Exemplary result for 6/10 patients where changes in methylation in the PBMC sample (red line) correlated with the changes in the content of CLL cells in the sample (blue line) as expected. Content of CLL cells was identifed from the percentage of CD5/CD19 double positive cells of all peripheral blood lymphocytes. Other panels: Four patients showed disproportional loss/gain of DNA-methylation, suggesting correlation of DNA-methylation with the clinical course of the disease. Right panel: In one patient, DNA-demethylation at 13q14.3 was directly associated with the clinical course of the disease. “P” = patient, “T” = therapy. “*” = progressive disease. (PDF) [file pgen.1003373.s003.pdf]

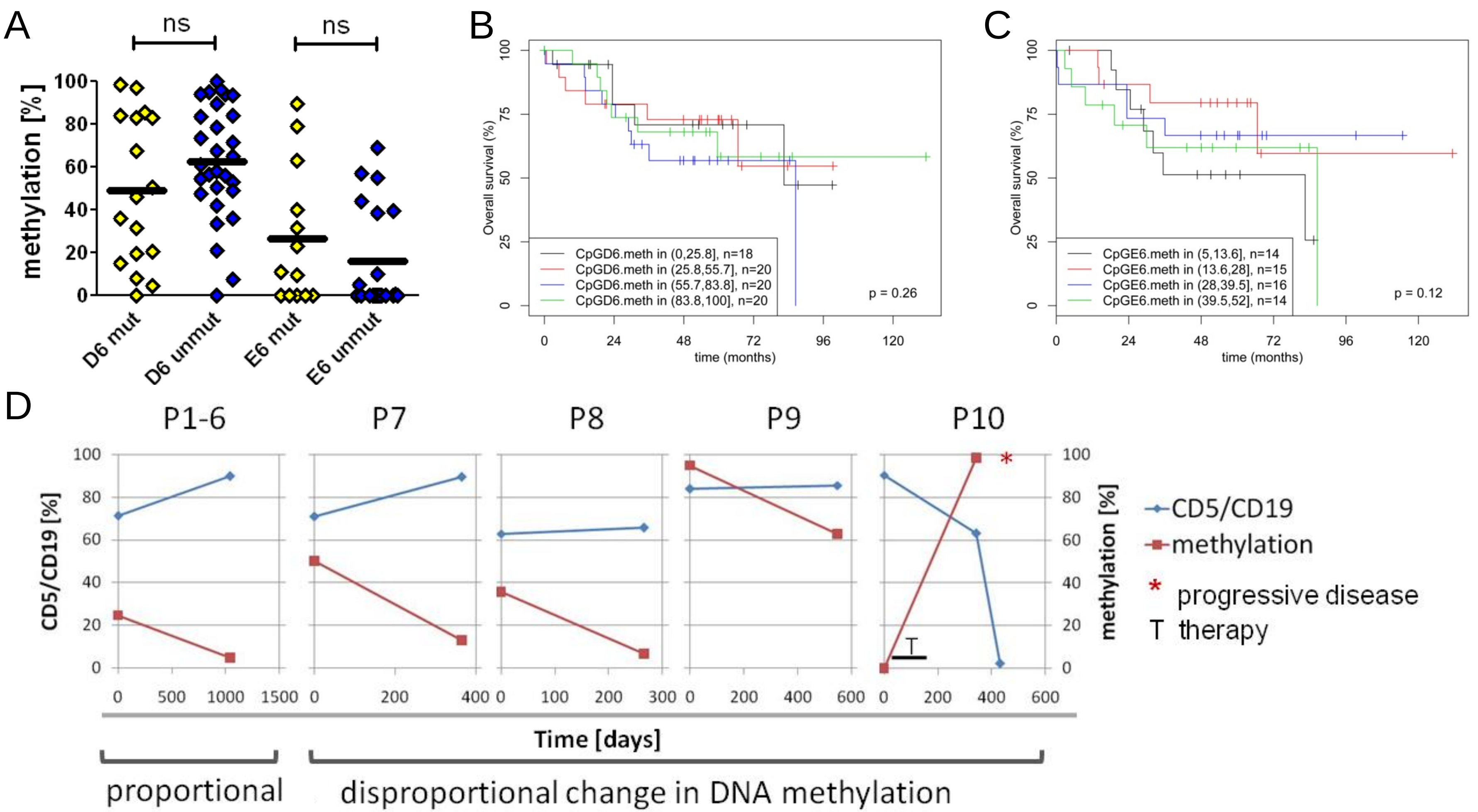

Figure S3. DNA methylation of 13q14.3 sequences does not correlate with IGHV mutations status and has no prognostic impact on overall survival, but changes during the clinical course of the disease.

(A) Scatterplots of methylation levels of CpG D6 and CpG E6 in the low risk patient group (normal karyotype and sole 13q deletion) comparing IGHV mutated samples with unmutated samples. There are no statistically significant differences in methylation levels between IGHV mutated and unmutated groups (D6 mutated: n=17, unmutated n=29; p=0.19 and E6, mutated: n=13, unmutated n=29; p=0.29, Wilcoxon rank sum test). (B, C) Kaplan-Meier estimates of overall survival of CLL patients (cohort composition see Table S1) comparing subgroups with different methylation levels (quartiles) of CpG D6 (A) and CpG E6 (B). In the analysed patient cohort, no statistically significant differences could be detected in overall survival distributions between subgroups (logrank test), suggesting that DNA-demethylation at 13q14.3 is common to all CLL. (D) Analysis of changes in DNA-methylation in D6 over time in CLL PBMC samples. Left panel: Exemplary result for 6/10 patients where changes in methylation in the PBMC sample (red line) correlated with the changes in the content of CLL cells in the sample (blue line) as expected. Content of CLL cells was identifed from the percentage of CD5/CD19 double positive cells of all peripheral blood lymphocytes. Other panels: Four patients showed disproportional loss/gain of DNA-methylation, suggesting correlation of DNA-methylation with the clinical course of the disease. Right panel: In one patient, DNA-demethylation at 13q14.3 was directly associated with the clinical course of the disease. “P” = patient, “T”= therapy. “*”=progressive disease.
