# Supplementary material for: Epigenetic Upregulation of lncRNAs at 13q14.3 in Leukemia Is Linked to the In Cis Downregulation of a Gene Cluster That Targets NF-kB
Source: PLoS Genet. 2013 Apr 4;9(4):e1003373. doi: 10.1371/journal.pgen.1003373 (PMC3616974; doi:10.1371/journal.pgen.1003373)
Supplement: Figure S4 — In vitro manipulation of DNA-methylation of D6 and E6 regions confirms functional relevance for gene expression. (Related to Figure 3.) (A, B) Haematopoetic and non-haematopoetic cell lines where tested for basal DNA methylation levels in D6/E6 using COBRA (A, n = 16) and MassARRAY analysis (B, n = 17) respectively. COBRA was controlled using in vitro methylated (“m”) and in vitro amplified non-methylated (“um”) Granta-519 genomic DNA as control and a plasmid containing several BstuI restriction sites. Jurkat, Raji and HEK cells carried methylation at both the D6 and E6 region. Only Jurkat cells showed full methylation at the D6 region and 70% methylation at the E6 region similar to B cells from healthy donors. Thus, only Jurkat cells were suited to study the impact of D6 and E6 methylation on the expression levels of 13q14.3 genes after treatment with 5-aza-2′-deoxycytidine. (C–F) DNA-demethylation of Jurkat cells in-vitro leads to an upregulation of 13q14.3 genes, but not of miRNA genes. Regions D6 (C) and E6 (D) that are differentially methylated in CLL patients and a CpG island reported to modulate RB1 expression (E) become demethylated in Jurkat cells upon 5-aza-2′-deoxycytidine treatment. (F) This demethylation leads to an increased expression of genes localized in the critical region with the exception of the miRNA genes that are post-transcriptionally regulated (Allegra, manuscript submitted). Gene expression was measured as in Figure 3. (G) The promoter of DLEU2/Alt1, the flanking CpG island E and the region E6 were cloned into the pCpGL luciferase vector. (H–J) Constructs depicted in G were either methylated in-vitro using SssI methylase (“m”) or left unmethylated (“um”) and subsequently transfected into HeLa, Granta519 and Mec1 cells. Promoter activity in HeLa was very low, suggesting that essential functional elements are missing in non-hematopoietic cells. In general, luciferase activity was lower than for the D6 constructs (Figure 4F and 4G), possibly [file pgen.1003373.s004.pdf]

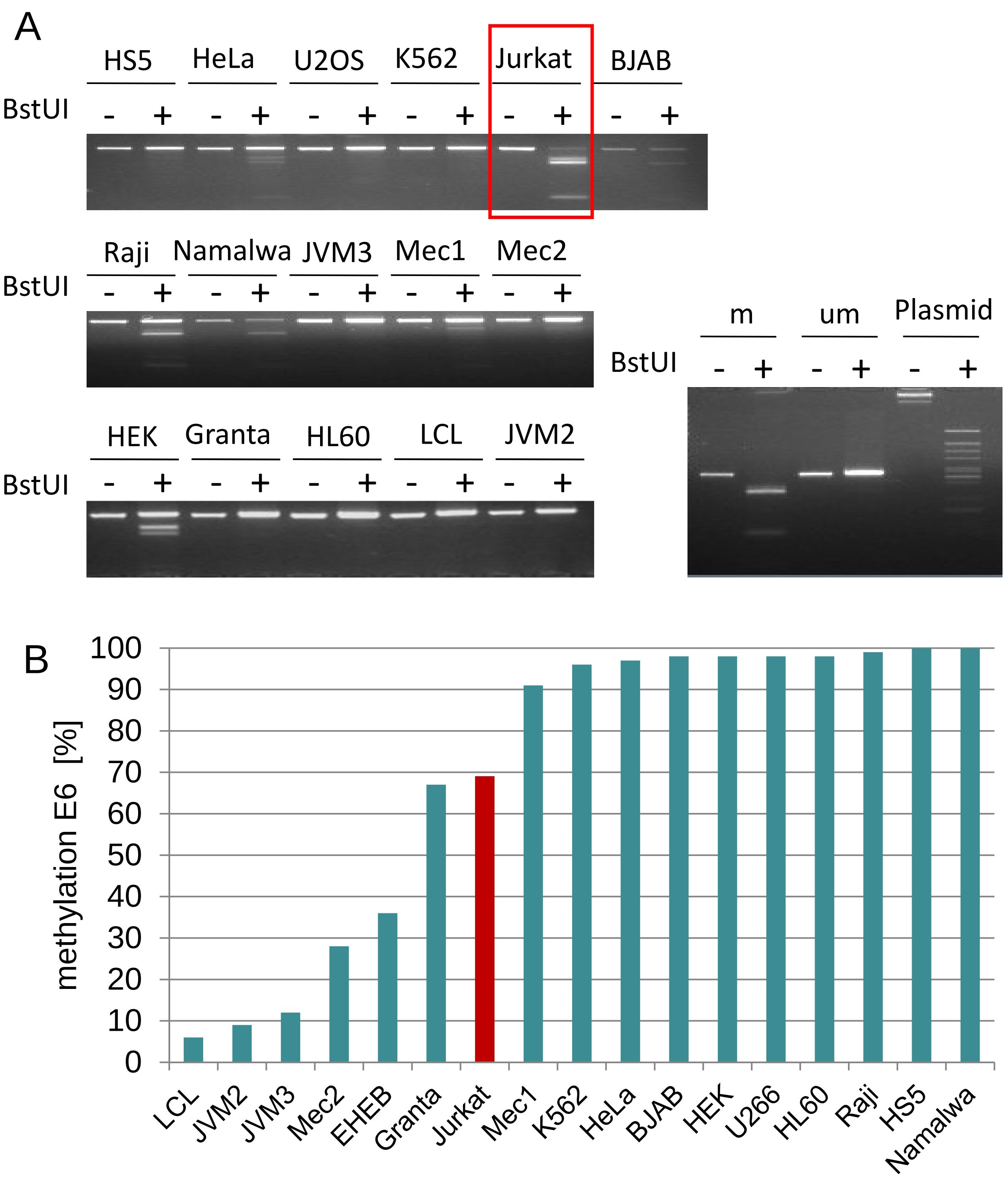

Figure S4 related to Figure 3: *In vitro* manipulation of DNA-methylation of D6 and E6 regions confirms functional relevance for gene expression.

(A, B) Haematopoetic and non-haematopoetic cell lines where tested for basal DNA methylation levels in D6/E6 using COBRA (A, n=16) and MassARRAY analysis (B, n=17) respectively. COBRA was controlled using *in vitro* methylated (“m”) and *in vitro* amplified non-methylated (“um”) Granta-519 genomic DNA as control and a plasmid containing several BstuI sites. Jurkat, Raji and HEK cells carried methylation at both the D6 and E6 region. Only Jurkat cells showed full methylation at the D6 region and 70% methylation at the E6 region similar to B cells from healthy donors. Thus, only Jurkat cells were suited to study the impact of D6 and E6 methylation on the expression levels of 13q14.3 genes after treatment with 5-aza-2'-deoxycytidine.

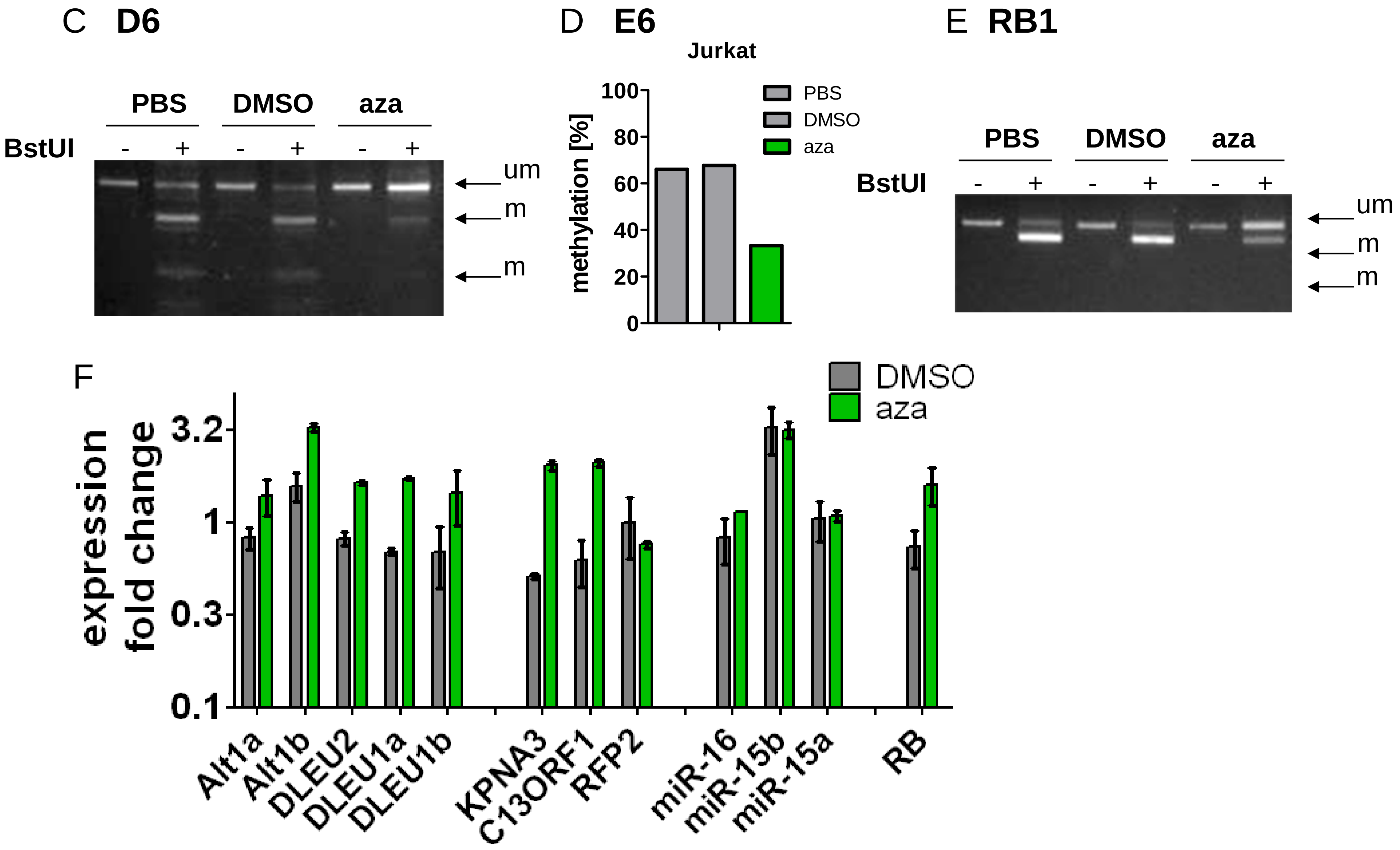

Figure S4 related to Figure 3: ***In vitro*** **manipulation of DNA-methylation of D6 and E6 regions confirms functional relevance for gene expression.**
(C-F) DNA-demethylation of Jurkat cells *in-vitro* leads to an upregulation of 13q14.3 genes, but not of miRNA genes. Regions D6 (C) and E6 (D) that are differentially methylated in CLL patients and a CpG island reported to modulate *RB1* expression (E) become demethylated in Jurkat cells upon 5-aza-2'-deoxycytidine treatment. (F) This demethylation leads to an increased expression of genes localized in the critical region with the exception of the miRNA genes that are post-transcriptionally regulated (Allegra, manuscript submitted). Gene expression was measured as in Figure 3.

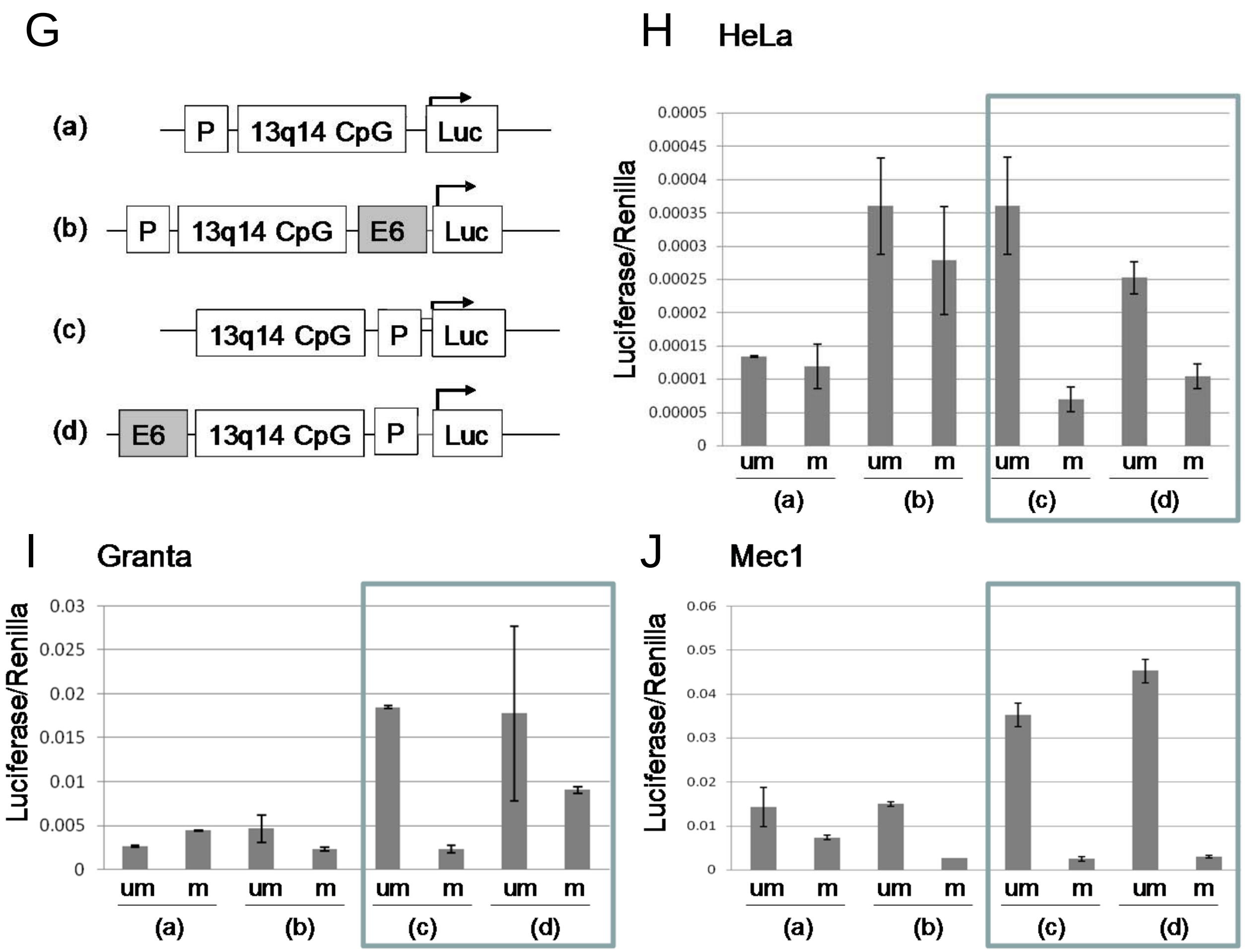

Figure S4 related to Figure 3: *In vitro* manipulation of DNA-methylation of D6 and E6 regions confirms functional relevance for gene expression.

(G) The promoter of *DLEU2/Alt1,* the flanking CpG island E and the region E6 was cloned into the pCpGL luciferase vector. (H-J) Constructs depicted in G were either methylated *in-vitro* using SssI methylase ("m") or left unmethylated ("um") and subsequently transfected into HeLa, Granta519 and Mec1 cells. Promoter activity in HeLa was very low, suggesting that essential functional elements are missing in non-hematopoietic cells. In general, luciferase activity was lower than for the D6 constructs (Figure 3 F, G), possibly because of the larger size of the constructs (7.9 and 7.4kbp (E6) vs 5.9 and 5.2kbp (D6)). Blue boxes mark constructs cloned in the physiological orientation.

K

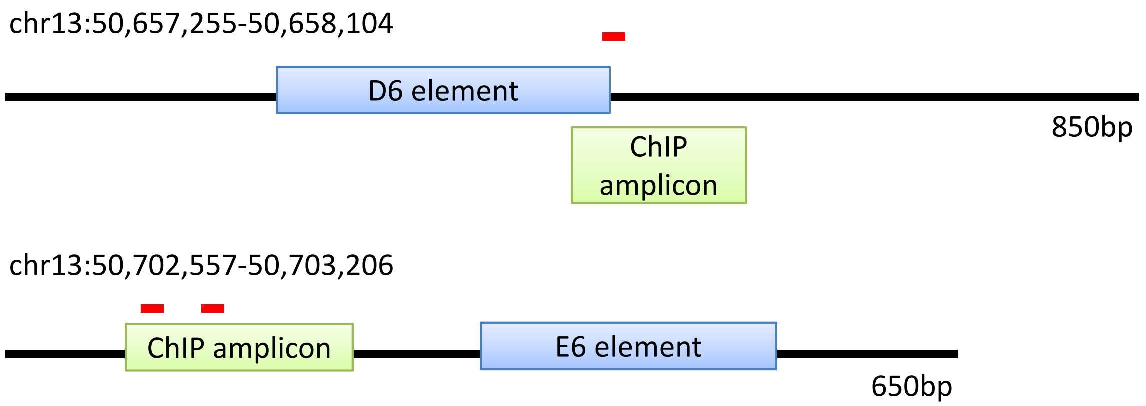

Figure S4 related to Figure 3: *In vitro* manipulation of DNA-methylation of D6 and E6 regions confirms functional relevance for gene expression.
(K) Schematic representation of regions analyzed for CTCF enrichment by ChIP-qPCR. Red lines represent predicted CTCF binding sites (http://bsproteomics.essex.ac.uk:8080/bioinformatics/ctcfbind.htm). Green boxes represent amplicons of ChIP qPCR and blue boxes represent D6 and E6 elements, respectively. Genomic locations are depicted on top of each panel.
