# Supplementary material for: Epigenetic Upregulation of lncRNAs at 13q14.3 in Leukemia Is Linked to the In Cis Downregulation of a Gene Cluster That Targets NF-kB
Source: PLoS Genet. 2013 Apr 4;9(4):e1003373. doi: 10.1371/journal.pgen.1003373 (PMC3616974; doi:10.1371/journal.pgen.1003373)
Supplement: Figure S5 — RNA–seq of chromatin-bound RNA shows no enhanced binding of DLEU1 and DLEU2 to chromatin. (Related to Figure 3). (A) In HeLa and U2OS cells, DLEU1 and DLEU2 do not show higher enrichment in the chromatin-bound RNA fraction when compared to the neighboring protein-coding genes and to total RNA. This suggests that they do not act via binding to chromatin. Localization of DLEU1 and DLEU2 is represented by the red box (top panel). Lines denote genes, blue boxes denote exons, arrows give direction of transcription. Blue bars represent numbers of reads, normalized to the highest peak whose number of reads is given at the left. (B) RNA-seq of chromatin-bound lncRNA genes used as controls. LncRNA reported to bind to chromatin show either specific enrichment in the chromatin-bound fraction (XIST and Tsix in mouse embryonic stem cells) or binding of specific sequences to chromatin (MALAT1 and TERC in HeLa and U2OS cells). (PDF) [file pgen.1003373.s005.pdf]

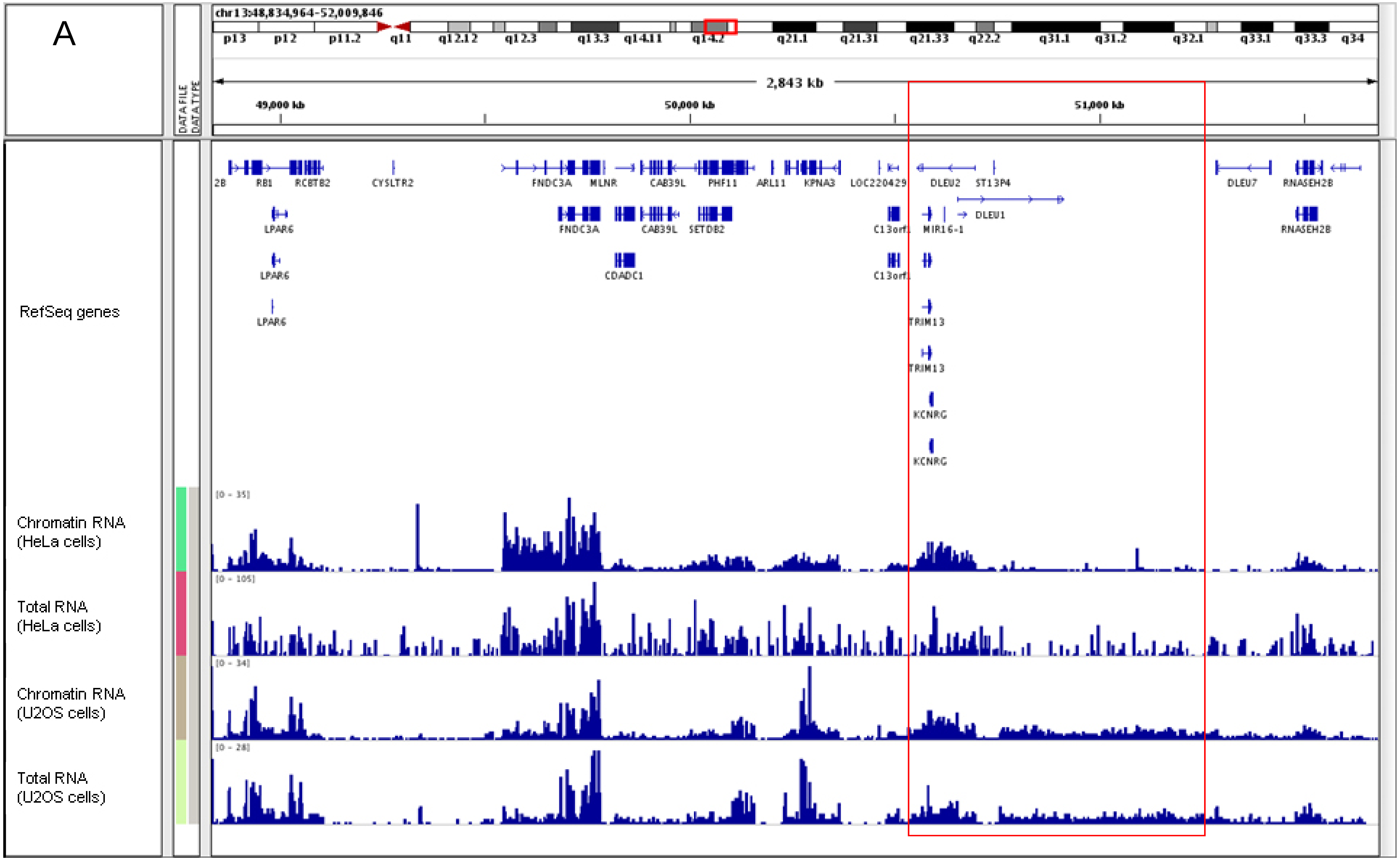

Figure S5 related to Figure 3: RNA-seq of chromatin-bound RNA shows no enhanced binding of *DLEU1* and *DLEU2* to chromatin.
(A) Localization of *DLEU1* and *DLEU2* is represented by the red box (top panel). Lines denote genes, blue boxes denote exons, arrows give direction of transcription. Blue bars represent numbers of reads, normalized to the highest peak whose number of reads is given at the left. In HeLa and U2OS cells, *DLEU1* and *DLEU2* do not show higher enrichment in the chromatin-bound RNA fraction when compared to the neighboring protein-coding genes and to total RNA. This suggests that they do not act via binding to chromatin.

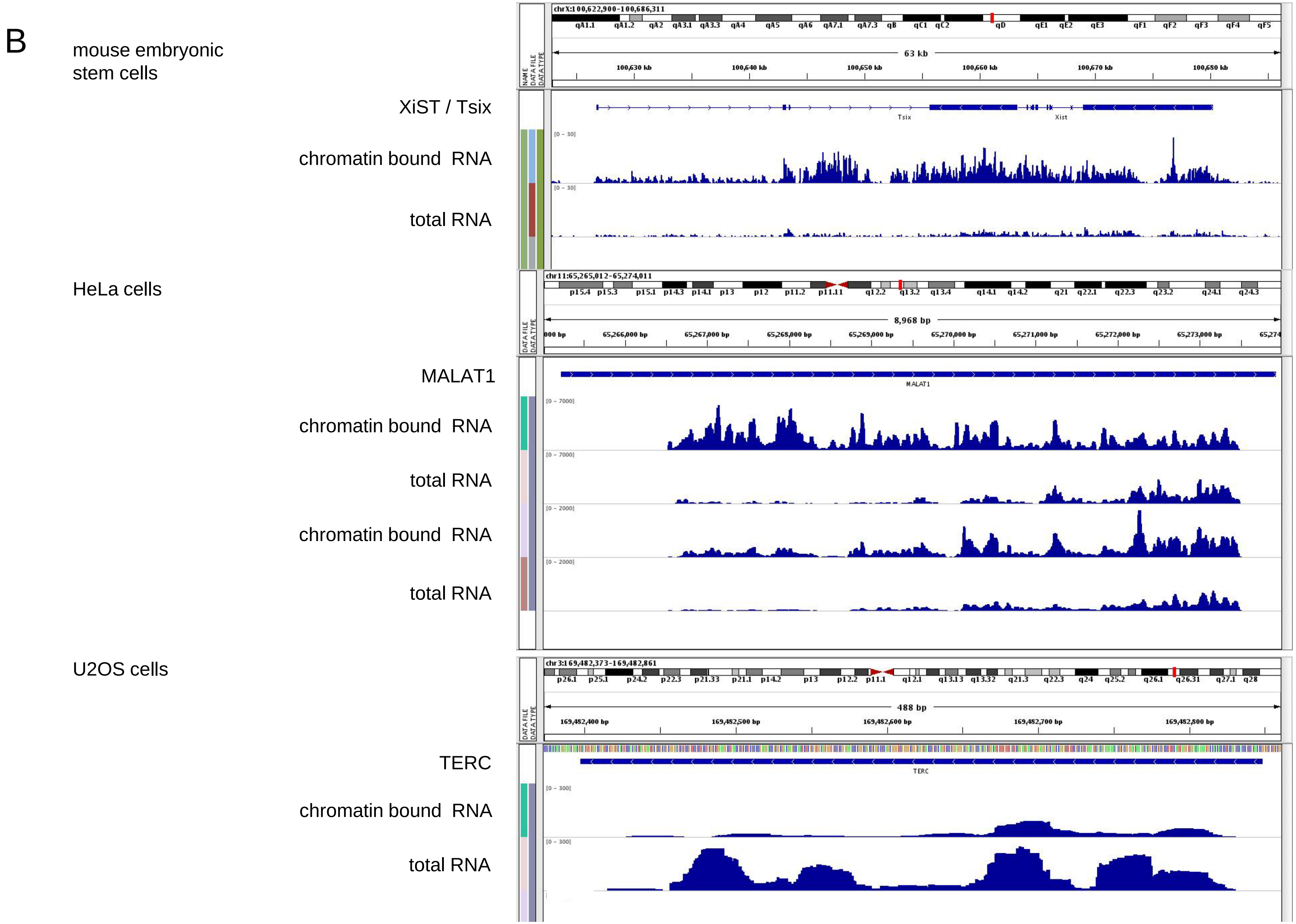

Figure S5 related to Figure 3: RNA-seq of chromatin-bound RNA shows no enhanced binding of *DLEU1* and *DLEU2* to chromatin.
(B) RNA-seq of chromatin-bound lncRNA genes used as controls. LncRNA reported to bind to chromatin show either specific enrichment in the chromatin-bound fraction (*XIST* and *Tsix* in mouse embryonic stem cells) or binding of specific sequences to chromatin (*MALAT1* and *TERC* in HeLa and U2OS cells).
