# Supplementary figures and images for: Epigenetic Upregulation of lncRNAs at 13q14.3 in Leukemia Is Linked to the In Cis Downregulation of a Gene Cluster That Targets NF-kB
Source: PLoS Genet. 2013 Apr 4;9(4):e1003373. doi: 10.1371/journal.pgen.1003373 (PMC3616974; doi:10.1371/journal.pgen.1003373)

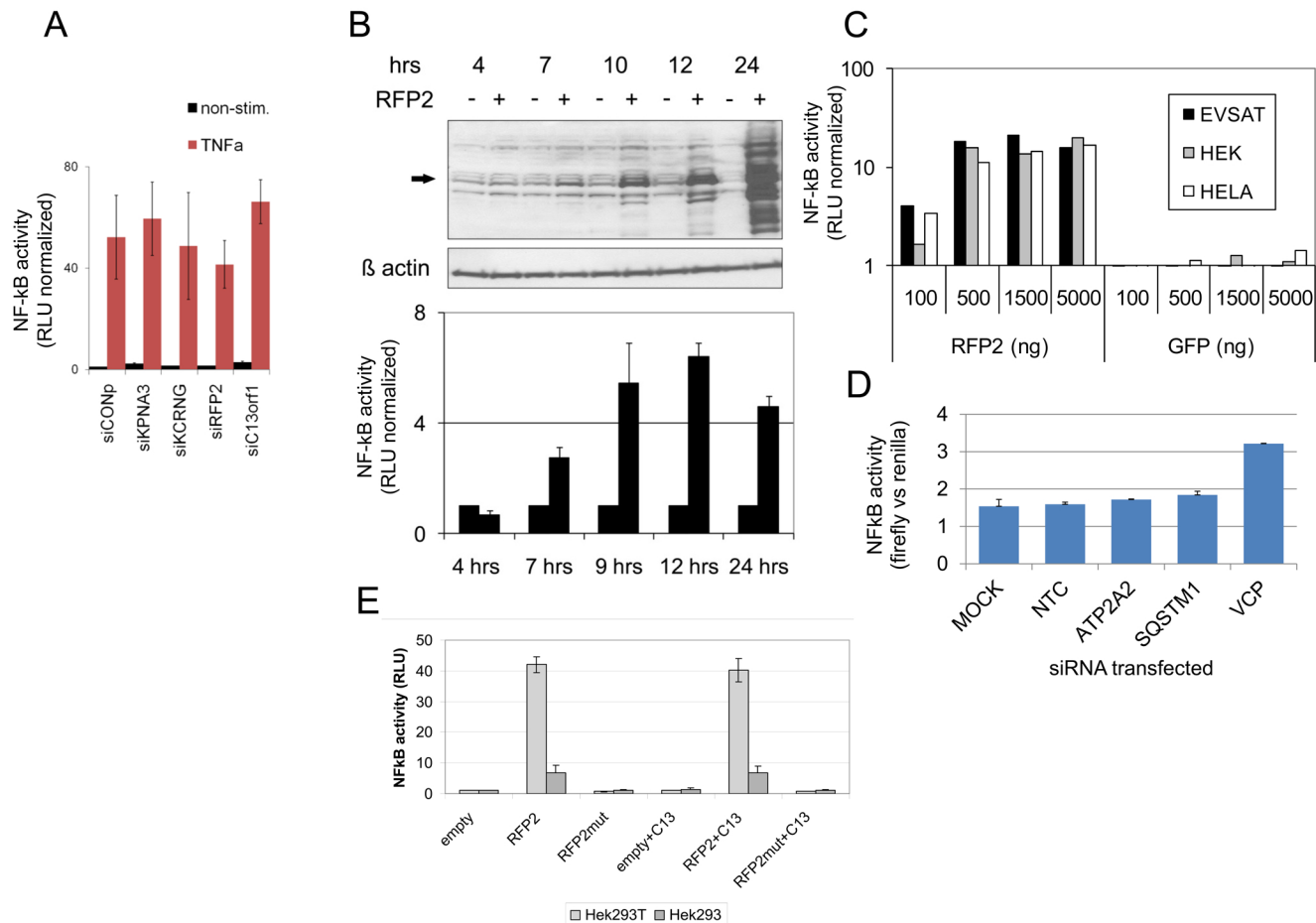

Figure S6 related to Figure 5: 13q14.3 candidate genes are involved in NF- $\kappa$ B signaling.

Supplement: Figure S6 — 13q14.3 candidate genes are involved in NF-kB signaling. (Related to Figure 4.) (A) Knockdown of 13q14.3 candidate tumor suppressor genes results in reduced activation of NF-kB by TNFa. KPNA3, KCNRG, RFP2 and C13ORF1 were knocked down in HEK293T cells and activity of NF-kB was measured after 24 hrs with a second reporter construct containing 5 synthetic NF-kB recognition sites. As negative control, a siRNA without physiological target was used (siCONP). Error bars signify SEM of 3 experiments. (B) RFP2 induces NF-kB activity in HEK293 cells. HEK293 cells were transiently transfected with CMV RFP2 expression plasmids (“+”) or empty vector (“−”) and NF-kB activity was measured after 4, 7, 10, 12 and 24 hrs (bottom panel). The top panel shows a representative Western blot of two experiments, error bars in bottom panel represent standard deviation of triplicate measurements. (C) Induction of NF-kB is not due to ectopic overexpression of protein. EVSAT, HEK293T and HeLa cells were transfected with increasing amounts of expression plasmids containing RFP2 or GFP. GFP-Fluorescence visualized by microscopy validated functionality and efficiency of transfection of the GFP plasmid (not shown). NF-kB activity was measured with luciferase reporter assay (for detailed description see Figure 6). Already 100 ng of RFP2 expression plasmid induced NF-kB activity, while no or little activity was induced with 5 µg of GFP expression plasmid. (D) Transfection of C13ORF1 expression plasmids into HEK293 and HEK293T cells alone or in combination with RFP2 or RFP2mut expression plasmids does not result in additional activation of NF-kB activity as measured by luciferase reporter assay. Error bars represent standard deviation of 3 independent experiments. (E) Knockdown of ATP2A2 and SQSTM1 does not modulate activation of NF-kB after transfection of RFP2, but knockdown of VCP substantially increases activation of NF-kB by RFP2. ATP2A2/SERCA2, SQSTM1 and VCP were knocked down and plasmids for [file pgen.1003373.s006.pdf]
