# Supplementary material for: Epigenetic Upregulation of lncRNAs at 13q14.3 in Leukemia Is Linked to the In Cis Downregulation of a Gene Cluster That Targets NF-kB
Source: PLoS Genet. 2013 Apr 4;9(4):e1003373. doi: 10.1371/journal.pgen.1003373 (PMC3616974; doi:10.1371/journal.pgen.1003373)
Supplement: Table S1 — Characteristics of CLL patient cohort (n = 143). (PDF) [file pgen.1003373.s007.pdf]

Table S1: Characteristics of CLL patient cohort (n=143)

**Hierarchical model of chromosome abnormalities**	**karyotype**	del 17p13	26
		del 11q22-23	19
		trisomy 12q13	3
		del 13q14	38
		normal	57
**IGHV**	**mutated**	47	
	**unmutated**	87	
	**NA**	9	
**Median age [years]**		62 (34-82)	
**Sex**	**male**	95	
	**female**	44	
	**NA**	4	
**Pretreated**	**yes**	23	
	**no**	90	
	**NA**	30	
**Binet stage**	**A**	43	
	**B**	19	
	**C**	22	
	**NA**	59	
**Analysis**	**aPRIMES**	29	
	**BioCOBRA/Sequenom**	82	
	**expression/MCIp/CTCF ChIP**	15-17	
	**histone ChIP**	5	
	**qRT-PCR**	28	
