# Supplementary material for: Epigenetic Upregulation of lncRNAs at 13q14.3 in Leukemia Is Linked to the In Cis Downregulation of a Gene Cluster That Targets NF-kB
Source: PLoS Genet. 2013 Apr 4;9(4):e1003373. doi: 10.1371/journal.pgen.1003373 (PMC3616974; doi:10.1371/journal.pgen.1003373)
Supplement: Table S2 — Characteristics of healthy donor cohort (n = 43). (PDF) [file pgen.1003373.s008.pdf]

Table S2: Characteristics of healthy donor cohort (n=43)

| | | |
|---|---|---|
| **Median age [years]** | | 56 (23-67) |
| **Sex** | **female** | 10 |
| | **male** | 27 |
| | **NA** | 6 |
| **Analysis** | **aPRIMES** | 9 |
| | **BioCOBRA/Sequenom** | 19 |
| | **expression/MCIp/CTCF ChIP** | 9 |
| | **histone CHIP** | 6 |
