# Supplementary material for: Epigenetic Upregulation of lncRNAs at 13q14.3 in Leukemia Is Linked to the In Cis Downregulation of a Gene Cluster That Targets NF-kB
Source: PLoS Genet. 2013 Apr 4;9(4):e1003373. doi: 10.1371/journal.pgen.1003373 (PMC3616974; doi:10.1371/journal.pgen.1003373)
Supplement: Table S3 — Transcription factors whose binding motifs are present in the D6 and/or E6 element were predicted using PATCH pattern search for transcription factor binding sites selecting a lower score boundary of 87.5. (PDF) [file pgen.1003373.s009.pdf]

Suppl. Table S3: Transcription factors whose binding motifs are present in the D6 and/or E6 element were predicted using PATCH pattern search for transcription factor binding sites selecting a lower score boundary of 87.5.

Specific to D6	Specific to E6	Present in both D6 and E6
AhR	C/EBPalpha	FXR
AR	C/EBPbeta	MyoD
Arnt	c-Ets-2	NF-1
C/EBPalpha	c-Myb	PPAR-alpha
C/EBPbeta	c-Myc	PPAR-gamma
C/EBPdelta	Crx	RAR-alpha1
Crx	EBP-80	RAR-beta
CTCF	Elk-1	RXR-alpha
Gbx2	FOXM1a	RXR-beta
HNF-1	GATA-1	RXR-gamma
HNF-1A	IPF1	SMAD-4
p53	Isl-1	Sp1
PITX2	JunB	SRY
RAR-gamma1	JunD	STAT1
SF-1	LEF-1	STAT5A
SMAD-3	RAR-gamma	STAT5B
Sp2	Sox-13	STAT6
Sp3	Sox-5	VDR
Sp4	SRF	
WT1-KTS	TBP	
YY1	TCF-1	
	USF2	
