# Supplementary material for: Epigenetic Upregulation of lncRNAs at 13q14.3 in Leukemia Is Linked to the In Cis Downregulation of a Gene Cluster That Targets NF-kB
Source: PLoS Genet. 2013 Apr 4;9(4):e1003373. doi: 10.1371/journal.pgen.1003373 (PMC3616974; doi:10.1371/journal.pgen.1003373)
Supplement: Table S4 — Primer sequences. (PDF) [file pgen.1003373.s010.pdf]

Table S4. Primer sequences

BioCOBRA

A_for	AACCCCAACTTAAAAAACCAC
Genomic	AACCCCAGCTTGGAGAACCAC
A_rev	GTTTTAGTTTTATATTTTTTTT
Genomic	GCCTCAGCTCCACATCCCCTCC
B_for	GAAGGGAGGGTTTTTATTTATGTGT
Genomic	GAAGGGAGGGCCCTTACCCATGTGC
B_rev	CCAACCTAAACTCAACCTACATTAC
Genomic	CCAGCCTAAGCTCAGCCTGCATTGC
C_for	GGGAGTTGTTTAGGTTAGTAGGGAT
Genomic	GGGAGTTGCCCAGGTCAGCAGGGAT
C_rev	AACAAATTTTCTAAACCACAAACAC
Genomic	AGCAGGTTTTCTGGACCACAGACAC
miR_for	TTTTTAGAAGATTAGATTTTTGT
Genomic	TCTTCAGAAGATCAGATCCTTGT
miR_rev	CTATACTAAACACAAAATAAACTTCAA
Genomic	CTGTGCTGGGCACAGAATGGACTTCAG
D4/5_for	TAAGTTGTTTTTAATTTTATTTTTT
Genomic	CAAGTTGCCCCTAACCCTACTTCCT
D4/5_rev	TAATACCTAATCTCATCAATCTAAC
Genomic	GCTAGATTGATGAGATCAGGCATCA
D6-for	GTTAGGTTGGAGTGTAGTGG
Genomic	GCCAGGCTGGAGTGCAGTGG
D6_rev	AAAAATTTTAAAATATTTACAACTTTATAA
Genomic	GAAGGTTTTAAAATATTTACAACTTTATAG
D7_for	GGTTGTTTAAAAATTAAATTATAGTTAGTG
Genomic	TGGTTGTTCAAAAATCAAACTACAGTTAGTG
D7_rev	TCATTTATCAATTTAATTTATCATTTTAAA
Genomic	TCATTTGTCAGTTTGATTTATCATTTTAAG
E5_for	GGTAGTTATTGTAGGGATAAAGG
Genomic	GGCAGTCACTGCAGGGACAAAGG
E5_rev	CAAACCAATCTACCAATATTAATTC
Genomic	CAGGCCAATCTGCCAGTGTTGGTTC
E7_for	TTTATTGGAATTAGTATTAGAAATTT
Genomic	CCCATTGGAACCAGCATCAGAAACCT
E7_rev	CACTACCAAAAACCCCAAAC
Genomic	CACTGCCAGAGACCCCAGGC
E7.2_for	TTTTTTAATTTTTTTTAGGTTGTTT
Genomic	TTTCCTAATCCCCTCTAGGCTGCCT
E7.2_rev	AACAAAAACTCTAACCTCACACCTC
Genomic	AGCAGAAACTCTGGCCTCACACCTC

MASSarray analysis

tag for mass array (small letters)

E6_for	aggaagagag
	GGAAGGTAGTTTTGGTTTTTTGTTA

Genomic	GGAAGGCAGTCTTGGTTTCCTGCTA
E6_rev	cagtaatacgactcactatagggagaaggct
	CCTAAAAATCTATTTCACCTATAACTC
Genomic	CCTAGGAGTCTATTTCACCTGTAGCTC
Rb_Sequenom_for	aggaagagag
	TTTTTAGTTGGGTGTTTTTTTGTG
Genomic	CCTCTAGCTGGGTGTTTTCCTGTG
Rb_Sequenom_rev	cagtaatacgactcactatagggagaaggct
	CCCCTACAACCCTTCAAAAAC
Genomic	CCCCTGCAGCCCTTCAGAAGC

Amplification from Topo vector backbone

M13_for	CGTTGTAAAACGACGGCCAGT
M13_rev	TTTCACACAGGAAACAGCTATGAC

Cloning D6/E6 into pCpGL

tag for restriction sites (small letters)

promoter CpG wo D6_for	aaactagtATTAAGTGAGAGTAGACCCC
promoter CpG wo D6_rev	aggatccTTCACTTATCACGCCATTAA
promoter CpG with D6_for	aaactagtATTTTTCGCGATTAAGTGA
promoter CpG with D6_rev	aggatccTTCTCCCACTTCCTTATTA
CpG promoter_for	aggatccATTAAGTGAGAGTAGACCCC
CpG promoter_rev	aaactagtTTCACTTATCACGCCATTAA
D6 CpG promoter_for	aggatccATTTTTCGCGATTAAGTGA
D6 CpG promoter_rev	aaactagtTTCTCCCACTTCCTTATTA
promoter CpG wo E6_for	aaactagtGCAGCGCCTCGTTCTATTTTTCC
promoter CpG wo E6_rev	aggatccTTTTGAGTCCTGTTAGTAAA
promoter CpG with E6_for	aaactagtGCAGCGCCTCGTTCTATTTTTCC
promoter CpG with E6_rev	aggatccCGTGCAGAATAAAGGCAACCACG
CpG promoter_for	aggatccGCAGCGCCTCGTTCTATTTTTCC
CpG promoter_rev	aaactagtTTTTGAGTCCTGTTAGTAAA
E6 CpG promoter_for	aggatccGCAGCGCCTCGTTCTATTTTTCC
E6 CpG promoter_rev	aaactagtCGTGCAGAATAAAGGCAACCACG

Cloning 3´UTRs of miR-15a/mir-16 target genes for luciferase reporter assays

3’UTR inserts of pMIR-Report containing restriction sites for SpeI, HindIII or SacI

Gene	Primer		Restriction site
TAB3	for:	TTTACTAGTCGCCAGGTTGAAAGTGAAAC	SpeI
	rev:	TTTAAGCTTGCAGTGTGCAACTTTGTCGT	HindIII
SMAD7	for:	TTTACTAGTAGCAGGCCACACTTCAAACT	SpeI
	rev:	TTTAAGCTTTCCTTTAGAAAAACATTCAGCTAGG	HindIII
SMRT(1)	for:	TTTACTAGTTCCGAGGACTGGACTGTTTT	SpeI
	rev:	TTTAAGCTTACACGCCTCTTGAACGGTAA	HindIII
CHUK	for:	TTTACTAGTTGTCCCCAAACCTATGGAAG	SpeI
	rev:	TTTGAGCTC TTTTCCTAAGCCATATTCAACTG	SacI

Gene expression 13q14

KPNA3_for	GCAAATCCAAAATTGCACTGT
KPNA3_rev	TGCATTGTCCAGCATCAGGT
C13ORF1_for	GGCAATCCAAAATTGCACTGT
C13ORF1_rev	TGCATTGTCCAGCATCAGGT
RFP2a_for	AAATACATCAGCTTGTAGGAGAC
RFP2a_rev	CCGTGGATCATCAAACAGACTACA
RFP2b_for	GGTTGGAGTTTGCGCATGCGTA
RFP2b_rev	TTTTGCAAAGCCGCGGAGGT
DLEU1a_for	TGCCGTTCTCCAGCGC
DLEU1a_rev	CCTCAGGTGTCCTCATCTGGTAA
DLEU1b_for	GCCTCGGAACAACTTTACCA
DLEU1b_rev	TCTCTTCCTTGCAACAATCTC
DLEU2_for	GATGCCTGATCTCATCAATCTAG
DLEU2_rev	AGGCTGTTCTCCAGAATTGG
Alt1a_for	CAGCGCCTCGTTCTATTTTT
Alt1a/b_rev	AACGGGGGTCTTCCTCCT
Alt1b_for	GAGGAAAAAGAAAGCCCACA
housekeeping genes	
cyc_for	GCTCGTGCCGTTTTGCA
cyc_rev	GCAAACAGCTCAAAGGAGACG
lmnb1_for	CTGGAAATGTTTGCATCGAAGA
lmnb1_rev	GCCTCCCATTGGTTGATCC
PGK_for	AAGTGAAGCTCGGAAAGCTTCTAT
PGK_rev	TGGGAAAAGATGCTTCTGGG
**miR expression 13q14**	
miR-16_for	TAGCAGCACGTAAATATTGGCGAA
miR-15a_for	TAGCAGCACATAATGGTTTGTGAAA
miR-15b_for	TAGCAGCACATCATGGTTTACAAAA
**CTCF ChIP controls**	
DM1_for	CTGGCCGAAAGAAAGAAATG
DM1_rev	CTCGAAGGGTCCTTGTAGCC
2kbup_DM1_for	CCCAGGTTGCCCTAAAAGTT
2kbup_DM1_rev	GCCTCTCCCTTCTCTGACG
**QPCR to control MCIP**	
SNRPN_for	TACATCAGGGTGATTGCAGTTCC
SNRPN_rev	TACCGATCACTTCACGTACCTTCG
ZAP70_amplicon2_for	CAGCTGGACAACCCCTACAT
ZAP70_amplicon2_rev	CGACCAGGAACTTGTGCAG
**MacroH2A/H3K4me2/CTCF ChIP**	
CpG-island A A1.2fwd	GCGGAAGCACACTCACTTC
A1.2rev	AGAACCCCAGCTTGGAGAAC
CpG-island A A2.1fwd	GACCTGGCGACTTTTACTGC
A2.1rev	GGCATTCTCCTCGTTCTCAC

CpG-island A A3.1fwd	GTGTCGCCCAGTTTCTTCAT
A3.1rev	TGCTGCCTGAAGGCTAAGAT
KPNA3 KPNA3e1.1_fwd	GCAGTAAAAGTCGCCAGGTCC
KPNA3e1.1_rev	GGGCTATACGTGCCCGTTG
KPNA3 KPNA3e3.1_fwd	AACAAAAGAGATGAACACTTATTGAAAAA
KPNA3e3.1_rev	AACATCTGAATCTTCTAGACTTTCTTCTTG
KPNA3 KPNA3e6.1_fwd	AAAACTGTTATCCAGTGACAGAAATCC
KPNA3e6.1_rev	TTGACTAGAATTGGTAAAATCCCAGAT
CpG-island B B2fwd	CTTGAACACAGAACCGGGCCCA
B2rev	ACGCTGCGCCGACACCATTT
CpG-island B B3fwd	CGCCCATTCGCGATGACAGACA
B3rev	TCGCAGATCTCTCCAGAAGCCG
CpG-island B B4fwd	CGGCATCTCCTTCAGAGGGATGTG
B4rev	ATGGCCACCTCGGTGTTGTGCT
C13ORF1 C13ORF1e2.1_fwd	GTTGTTATTGTAAAGAATGGAAGAAGAATATGT
C13ORF1e2.1_rev	TGTAAAGGTGCGCTGGCTAAA
C13ORF1 C13ORF1e3.1_fwd	AATCAGATTCCTCTTGGCCGA
C13ORF1e3.1_rev	GCTCCATCATTTCTCATCACCAG
C13ORF1 C13ORF1e4.1_fwd	TGACCATGTCGAATTAAATGTATACTTGA
C13ORF1e4.1_rev	CCCTCGTATACCTGATGCTGGA
C13ORF1 C13ORF1e5.1_fwd	TCATGACCCTGAGTCTTTGCCT
C13ORF1e5.1_rev	GGGCGTTAACTAAAGGACAGTGATT
CpG-island C C1fwd	CGGAGCCGCGAGTCCATTTT
C1rev	TTCGAGGACCACCCCGCTTT
CpG-island C C2fwd	CAGAAACCAGCGGGGCACTGTCAT
C2rev	CGGACGGAGCAGGTTTTCTGGA
CpG-island C C3fwd	GCTACCAGCGTCTCCACATCCCCTA
C3rev	ATGCAACCAAACGCTGGCGG
CpG-island C C1a_fwd	TAGAATGGGAGGCAGGTTTG
C1a_rev	CCGTTGACTAATAATGCTTAGCC
CpG-island C C2a_fwd	GGCTAAGCATTATTAGTCAACGG
C2a_rev	CGTTCCTTCCGGCGTCTC
CpG-island C C3a_fwd	GAGACGCCGGAAGGAACG
C3a_rev	CCTACAAGCTGATGTATTTGGCA
CpG-island C C4a_fwd	TGCCAAATACATCAGCTTGTAGG
C4a_rev	GATGGGATTTCACCATATTGGTC
CpG-island C C5a_fwd	GACCAATATGGTGAAATCCCATC
C5a_rev	TAGGTTTAACAGGCTCTCTAGTGAAG
CpG-island C C6a_fwd	CTTCACTAGAGAGCCTGTTAAACCTA
C6a_rev	ATAATCACATCAGCAAGAAATCAC
RFP2 RFP2e1.1fwd	GTACCGTGCGGTCCCTGTAGTTGGA
RFP2e1.1rev	CTAGTGGGAAAGGCCGCGCG
RFP2 RFP2e1.2fwd	TACCGTGCGGTCCCTGTAGTTGGA
RFP2e1.2rev	TCGCTGAAGGTTCGAGGACCAC
RFP2 RFP2e2.1fwd	TCGCTGAAGGTTCGAGGACCAC
RFP2e2.1rev	GCTGATGTATTTGGCAGGGT
RFP2 RFP2e3.1fwd	TGCTTGAAGAAGATCTCACATGCCC
RFP2e3.1rev	GGAATTCCGCACACTCCCTTCTAAG

RFP2 RFP2e3.2fwd	TGGAGACCAGCTCCATTCAAGTGTC
RFP2e3.2rev	TGCATACTGGCATTTTGGGAGAGAT
CpG-island D D1fwd	GCAGCCAGGGCTTGGGAAACTCTT
D1rev	CCGCCTTTTCTCGCCGTTTT
CpG-island D D2fwd	ACAGGTTATCCTGTCTCTCCCGCT
D2rev	CGGGGTTGGCTCTAACGAATTT
CpG-island D D3fwd	CAGACGCCCAAGTTGCCCCTAA
D3rev	GGCGCGGGGTCTACTCTCACTTAAT
CpG-island D D4fwd	TCCCGAGTCCCGCTCTGCTACTTCT
D4rev	TACTTGGAGCAAAGGGCAGTCGGC
CpG-island D D5fwd	GGCTGCCTCCACAGCTGTCAATACC
D5rev	GGTTGGAGTTTGCGCATGCGTA
CpG-island D D1a_fwd	AAATTAGTTGCCTGGCATACAATG
D1a_rev	CTTCAAATTTGAGAATACACTAGCG
CpG-island D D2a_fwd	CGCTAGTGTATTCTCAAATTTGAAG
D2a_rev	TGCTAATTCAGTAACTGCAGTGAAC
CpG-island D D3a_fwd	GTTCACTGCAGTTACTGAATTAGCA
D3a_rev	CTGGTGGTTTCTATCAATTCTTACTC
CpG-island D D4a_fwd	GAGTAAGAATTGATAGAAACCACCAG
D4a_rev	GGTCTACTCTCACTTAATCGCGA
CpG-island D D5a_fwd	TCGCGATTAAGTGAGAGTAGACC
D5a_rev	GGTTGAATCGTACAGCGTGGT
CpG-island D D6a_fwd	ACCACGCTGTACGATTCAACC
D6a_rev	TGCTACCCTTCTCCCACTTC
CpG-island D D7a_fwd	GTGCCTGAAGTACTAATAAGGAAGTG
D7a_rev	TAACATCCTTATTTGCCTAACAGG
BCMS BCMSe1.1fwd	TTTTGCAAAGCCGCGGAGGT
BCMSe1.1rev	GGTAGCTATAAGACGACCCCTCGGC
BCMS BCMSe1.2fwd	GCCCACAGGCATTTAGTCTACGTTG
BCMSe1.2rev	TTTCTTTTCTCCTAAGCAGGACCCG
BCMS BCMSe2.1fwd	ATGAGGACACCTGAGGTTCAGA
BCMSe2.1rev	TTCTAAGACTTTGGGGCAGA
BCMS BCMSe3.1fwd	CCTTCAGGAATTGAGTCACAATGCA
BCMSe3.1rev	AAAAGGGAAAGAATGGCTGGCAA
BCMS BCMSe3.2fwd	TCTGGCAATAATAAAGTTGGGCAGC
BCMSe3.2rev	GGAAAGGGCCAAGAAACTGCTGT
CpG-island E E1fwd	TACCTCCTGGATTTACAAACTGGG
E1rev	TCTTCAACAGTGTATAAATGCTACACAAAC
CpG-island E E1.1fwd	CTTTCAGTTGTTGCCTCCAAACGG
E1.1rev	CGCGGTGAGCGCTATTTATAGGTAG
CpG-island E E12fwd	GCGCCTCGTTCTATTTTTCCT
E12rev	GGTCAGTCCGTACCGCCC
CpG-island E E1.2fwd	GAACCCCCCGGCTCGATTTT
E1.2rev	TCCGCCTTCTCCTTTTCGCAA
CpG-island E E2fwd	GGGAGTTTGCAGACCGGAG
E2rev	TCGTCATAAGGCTTTGAAGGAAAG
CpG-island E E3fwd	CAGCACTCCTAAGACGGCG
E3rev	TGCAGCTTGGCGCGA

CpG-island E E4fwd	AATCTGGTCAGCTTAAAAGTGTCCTC
E4rev	TGGACTCATTTAGGATCACCAGAT
CpG-island E E5fwd	GTTCAGACTTCAGTACCTAAAACGTTATG
E5rev	GACTCTAAATCGAGGTTTCTGATGCT
CpG-island E E6fwd	CGCTAGTCTAGCCCAGCGT
E6rev	TCCTAACTCTTTGGGTCCCTGTAA
CpG-island E E7fwd	CTGCTACAGCAGGCTTCGC
E7rev	AGTGCTACAAACTCTTTCTCCATTTTCT
CpG-island E E8fwd	ACTCCAGTGGTCTGCAAGCC
E8rev	GGCAAGCGATTGATTCCG
CpG-island E E9fwd	GTGCATCCTGGTTAGAATTGGG
E9rev	GGCAAGCGATTGATTCCG
CpG-island E E10fwd	AGGGAATTCTGTATTCTAGTTGTGGATCT
E10rev	TTGAACGACCTTGGGCCA
CpG-island E E11fwd	TGTTAACTCAAGGCCGATTACCTT
E11rev	TTCCCTCCACACTCTTCCTACA
CpG-island E E1a_fwd	TCTCTCTTAACATCTACCTCCTGGAT
E1a_rev	GACGCGTCCCATTGTGTGA
CpG-island E E2a_fwd	TCACACAATGGGACGCGTC
E2a_rev	GACAGGTCAGTCCGTACCGC
CpG-island E E3a_fwd	GCGGTACGGACTGACCTGTC
E3a_rev	TCTCACATCTAACTTCCAGTAACTTCTC
CpG-island E E4a_fwd	GAGAAGTTACTGGAAGTTAGATGTGAGA
E4a_rev	CGCCGTCTTAGGAGTGCTG
CpG-island E E5a_fwd	CAGCACTCCTAAGACGGCG
E5a_rev	TGGACTCATTTAGGATCACCAGAT
CpG-island E E6a_fwd	ATCTGGTGATCCTAAATGAGTCCA
E6a_rev	TAGGTACTGAAGTCTGAACCTAGCC
CpG-island E E7a_fwd	GGCTAGGTTCAGACTTCAGTACCTA
E7a_rev	AGACGTGCAATAGCCACCG
CpG-island E E8a_fwd	CGGTGGCTATTGCACGTCT
E8a_rev	ACGCTGGGCTAGACTAGCG
CpG-island E E9a_fwd	CGCTAGTCTAGCCCAGCGT
E9a_rev	GCGAAGCCTGCTGTAGCAG
CpG-island E E10a_fwd	CTGCTACAGCAGGCTTCGC
E10a_rev	GGCAAGCGATTGATTCCG
CpG-island E E11a_fwd	CGGAATCAATCGCTTGCC
E11a_rev	TTCCCTCCACACTCTTCCTACA
CpG-island E E12a_fwd	CGGAATCAATCGCTTGCC
E12a_rev	CAAGGATACTGAAGTTCTTGTAAGTAGC
BCMSUN BCMSUNe2.2fwd	TCTCATGTCATGTTCCAATGCA
BCMSUNe2.2rev	GGAGAACAGCCTCACTTCTTTGA
BCMSUN BCMSUNe3.1fwd	TTGTAAAAACGGATGGGTGCA
BCMSUNe3.1rev	TCGATGCTGCTTGTCAGCTG
BCMSUN BCMSUNe3.2fwd	CTTTGGCCACCATTATTGCATATT
BCMSUNe3.2rev	ATCGATGCTGCTTGTCAGCTGt
BCMSUN BCMSUNe4.2fwd	GACCAGAAAGCAACTATATGATTACCTTT
BCMSUNe4.2rev	AAAGTTTGACATTTGCAATAGCATTC

controls

GAPDH GAPDHprom_fwd	CCGGGATTGTCTGCCCTAAT
GAPDHprom_rev	GCACGGAAGGTCACGATGT
MYH1 MYH1prom_fwd	ACCACGATTTCAGCAAGAATG
MYH1prom_rev	GAGCAGGAAAATCGCAGAAC
CDH12 CDH12a96F04_fwd	TGCCATGTGGAGATGAGAAG
CDH12a96F04_rev	ACCCAACATTCCACCAAATG
CDH12 CDH12b96F04_fwd	GACCACTGAAAAGAAAGGCATTA
CDH12b96F04_rev	ACCCAACATTCCACCAAATG
E6ChIP_CTCF_for1	TCTGTGCTTCCTCTCACTGG
E6ChIP_CTCF_rev1	AGGCTAAACAATGCAAACAGG
D6CHIP_CTCF_FWD1	GTTGGGATTACAGGCGTGAG
D6CHIP_CTCF_REV1	GGGACTTCCTTATTAGTACTTCAGG

NF-kB target genes used after transfection of miR15a and miR16

Primer name	Sequence	UPL Probe number
GAPDH_L	AGCCACATCGCTCAGACAC	#60
GAPDH_R	GCCCAATACGACCAAATCC	#60
HPRT-1_L	TGACCTTGATTTATTTTGCATACC	#73
HPRT-1_R	CGAGCAAGACGTTCAGTCCT	#73
IL6-L	GCCCAGCTATGAACTCCTTCT	#45
IL6-R	GAAGGCAGCAGGCAACAC	#45
IL8_L	AGACAGCAGAGCACACAAGC	#72
IL8_R	ATGGTTCCTTCCGGTGGT	#72
CXCL1_L	TCCTGCATCCCCCATAGTTA	#52
CXCL1_R	CTTCAGGAACAGCCACCAGT	#52
