# Supplementary material for: Epigenetic Upregulation of lncRNAs at 13q14.3 in Leukemia Is Linked to the In Cis Downregulation of a Gene Cluster That Targets NF-kB
Source: PLoS Genet. 2013 Apr 4;9(4):e1003373. doi: 10.1371/journal.pgen.1003373 (PMC3616974; doi:10.1371/journal.pgen.1003373)
Supplement: Table S5 — miRNA families tested for induction of NF-kB, ranked according to their induction of NF-kB. (Related to Figure 4.) (PDF) [file pgen.1003373.s011.pdf]

Table S5 related to Figure 4. miRNA families tested for induction of NFkB, ranked according to their induction of NFkB

MiRNA Family	Members
miR15/16/195/424/497	miR-15a, miR-15b, miR-16, miR-195, miR-424, miR497
miR-10	miR-10a, miR-10b
miR-27ab	miR-27a, miR-27b
miR-181	miR-181a, miR-181b, miR-181c, miR-181d
miR–500/501/502/502	miR-501-3p, miR-502-3p
miR–507/557	miR-507, miR-557
miR–29abc	miR-29a, miR-29b, miR29c
miR–141/200a	miR-141, miR-200a
miR-196ab	miR-196a, miR-196b
miR-204/211	miR-204, miR-211
miR–26ab	miR-26a, miR-26b
miR–548a/548b/548c/548d/548hij/559	miR-548d-5p, miR-548c-5p, miR-548b-5p, miR-548a-5p, miR-559
miR–200bc/429	miR-200b, miR-200c, miR-429
miR–17/20/93.mr/106/519.d	miR-17-5p, miR-20a, miR-20b, miR-93, miR-106b, miR-106a, miR-519d
miR-23ab	miR-23a, miR-23b
miR518d/519b/519c/520c/526a	miR-518d-5p, miR519b-5p, miR-519c, miR-520c-5p, miR-526a
miR-18ab	miR-18a, miR-18b
miR518a/527	miR-518a-5p, miR-527
miR-520gh	miR-520h, miR-520g
miR-19	miR-19a, miR-19b
miR–135	miR-135a, miR-135b
miR–130/301	miR-130b, miR-130a, miR-301, miR-301b
miR450b/769	miR-450b-3p, miR-769-3p
miR509/509	miR-509-3-5p, miR-509-5p
let-7/98	let-7a, let-7b, let-7c, let-7d, let-7e, let-7f, let-7g, let-7i, miR-98
miR–132/212	miR-132, miR-212
miR–518a/518bcf/518d	miR-518b, miR-518c, miR-518f
miR-1/206	miR-1, miR-206, miR-613
miR–515/519e	miR-515-3p, miR-519e
miR–517ac	miR-517a, miR-517c
miR–106/302	miR-302a, miR-302b, miR-302c, miR-302d, miR-372, miR-373, miR-520b, miR-520c
