# Supplementary material for: Epigenetic Upregulation of lncRNAs at 13q14.3 in Leukemia Is Linked to the In Cis Downregulation of a Gene Cluster That Targets NF-kB
Source: PLoS Genet. 2013 Apr 4;9(4):e1003373. doi: 10.1371/journal.pgen.1003373 (PMC3616974; doi:10.1371/journal.pgen.1003373)
Supplement: Table S6 — Mann-Whitney Rank Sum Test for differential methylation. (p-values). (PDF) [file pgen.1003373.s012.pdf]

Suppl. Table S6: Mann-Whitney Rank Sum Test for differential methylation. (p-values)

	D6	E6
young vs old	0,9681	0,9522
non-del(13q) vs del(13q)	0,9761	0,4965
