# Supplementary material for: Epigenetic Upregulation of lncRNAs at 13q14.3 in Leukemia Is Linked to the In Cis Downregulation of a Gene Cluster That Targets NF-kB
Source: PLoS Genet. 2013 Apr 4;9(4):e1003373. doi: 10.1371/journal.pgen.1003373 (PMC3616974; doi:10.1371/journal.pgen.1003373)
Supplement: Figure S2 — Details of the transcriptional start sites of DLEU1 (A), DLEU2/Alt1 (B) and the homologous region on 3q25.33 (C), (D) a comparison of 13q14 and 3q25 and details of RB1 (E) and DLEU7 (F). (Related to Figure 1.) (A) D6 is hypomethylated in CLL patients compared to non-malignant B-cells as measured by aPRIMEs and BioCOBRA. MCIp for unknown reasons did not faithfully represent hypomethylation detected by aPRIMES and BioCOBRA. (A, B) For details see legend to Figure 1. (C) At the homologous region in chromosomal band 3q26 that harbors miR-15b and miR-16-2, TRIM59 and KPNA4, DNA-methylation was measured by MCIp and transcriptional activity by gene expression profiling. Only minor differences could be detected between CLL samples and non-malignant B-cells. Positions refer to genome build hg18. (D) Regions of 13q14.3 and 3q25.3 have similar coding potential. Performing pairwise alignment of all protein coding genes from chr13:50,139,149–51,673,000 and chr3:158,760,141–160,445,393, a striking protein sequence similarity of a subset of protein coding genes (green boxes) was found. TRIM proteins: 57% similarity, e value: 1e-65; ARL proteins: 62% similarity, e value: 2e-53; KPNA proteins 92% similarity, e value: 0. Similarly, sequence alignment of hsa-mir-16-1 and hsa-mir-16-2 showed 68,9% identity of a 90 bp overlap and alignment of hsa-mir-15a and hsa-mir-15b showed 56,1% identity of a 98 bp overlap. It can therefore be speculated that both regions arose from duplication and inversion. “cen” centromeric, “tel” telomeric. (E) DNA-methylation was measured at an internal CpG island reported to correlate with imprinting of RB1 by regulating transcription of an alternative transcript [33]. Differences in DNA-methylation can only be observed in patients with del13q, possibly caused by gene dosage effects. (E) The median log 2 fold methylation of 12 tiling array oligos spanning the CpG island at the transcriptional start site of DLEU7 (chr 13:50315373–50316150, hg18) were compared a [file pgen.1003373.s002.pdf]

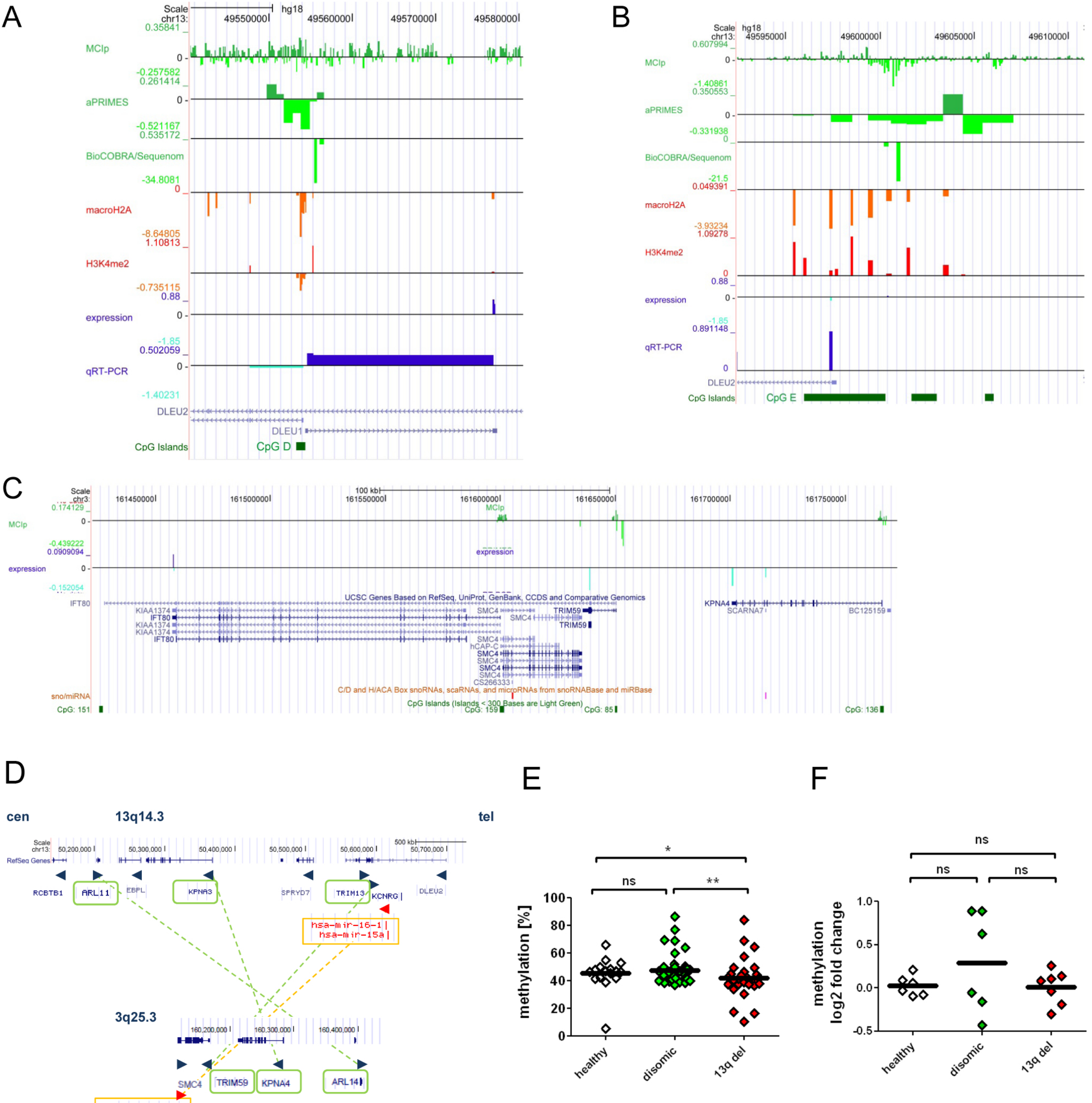
A
B
C
D
E
F
MCIp
aPRIMES
BioCOBRA/Sequenom
macroH2A
H3K4me2
expression
qRT-PCR
DLEU2
DLEU1
CpG Islands
CpG D
CpG E
UCSC Genes Based on RefSeq, UniProt, GenBank, CCDS and Comparative Genomics
C/D and H/ACA Box snoRNAs, scaRNAs, and microRNAs from snoRNABase and miRBase
CpG Islands (Islands < 300 Bases are Light Green)
cen
13q14.3
tel
3q25.3
RCBTB1
ARL11
EBPL
KPNA3
SPRYD7
TRIM13
KCNRG
DLEU2
hsa-mir-16-1
hsa-mir-15a
SMC4
TRIM59
KPNA4
ARL14
hsa-mir-15b
hsa-mir-16-2
methylation [%]
healthy
disomic
13q del
ns
*
**
methylation log2 fold change
Figure S2
